# Mathematical Model on the optimal control of HIV/AIDS transmission dynamics, incorporating the uninfected class after an effective exposure to the disease

**DOI:** 10.1371/journal.pone.0346773

**Published:** 2026-04-15

**Authors:** Christopher Chukwuma Asogwa, Stephen Ekwueme Aniaku, Obiageri Mary Joy Ezugorie, Emmanuel Mbah

**Affiliations:** 1 Department of Mathematics, University of Nigeria, Nsukka, Nigeria; 2 Department of Mathematics, University of Ilorin, Ilorin, Kwara State, Nigeria; Gulf University for Science and Technology, KUWAIT

## Abstract

In this work, we formulated a mathematical model on HIV/AIDS transmission dynamics that incorporates the post-contact control measure. The six important compartments of the model are: Susceptible, under-3-day Exposed, Exposed-Uninfected, Treatment, Pre-AIDS, and AIDS. The next-generation operator approach was used to obtain the effective reproduction number $\left(R_{e}\right)$. From the stability analysis, it was established that the disease-free equilibrium point of the model is locally asymptotically stable when the reproduction number is less than one $\left(R_{e}<1\right)$, and that it has a unique endemic equilibrium whenever this number exceeds unity. In the optimal control model, both Pre-contact Preventive and Anti-retroviral therapy (ART) control measures were incorporated. Pontryagin’s maximum principle was used to form an optimal system. Seven control strategies were considered. The most cost-effective control measure was identified to be ‘the optimal application of Pre-contact preventive control measures’. In double control strategies, ‘The combined use of Pre-contact Preventive and Anti-retroviral therapy’ was also found to be better and most cost-effective than the other double control strategies in controlling the virus. The population dynamics of different classes with respect to the application of each of these strategies were simulated with MATLAB to assess the impact of these control measures on the control of the virus.

## 1. Introduction

Since its detection in the 1980s, the Human Immune-deficiency virus (HIV), the causative agent of the acquired immune deficiency syndrome (AIDS), has continued to pose major public health and socioeconomic challenges globally [1,2]. As of 2022 Approximately 39.0 million [33.1–45.7 million] people were living with HIV at the end of 2022, with many countries reporting an increasing trend in new infections [3]. Also, as of the end of 2024, it was estimated that 40.8 million people were living with HIV, and 65% of them are in the African Region [4]. AIDS, which is the advanced stage of HIV infection, is considered a pandemic disease that is actively spreading. Sub-Saharan Africa has been identified as the region that carries the vast majority of the global disease burden worldwide [5].

Genetic research indicates that HIV originated from West Africa during the twentieth century [6]. In the first few weeks of its infection, which occurs in 40–90% of cases, symptoms may include fever, large tender lymph nodes, throat inflammation, rashes, headache, and sores of the mouth and genitals. Some people also develop other opportunistic infections like Tuberculosis (TB). Also, gastrointestinal symptoms such as vomiting and diarrhea may occur.

HIV is transmitted primarily via unprotected sexual intercourse, contaminated blood transfusions, and from mother to child during pregnancy, delivery, or breastfeeding [7]. It can also be transmitted through contaminated sharps, such as needles, syringes, blades, and other surgical instruments. Sharing or reusing contaminated needles and syringes among drug users is a risk factor for HIV transmission. Circumstances associated with needle stick injuries included unexpected patient movement, handling or disposal of used needles, needle recapping, accidental stick by a colleague, and needle disassembly. However, the virus does not live long or replicate outside the body or on surfaces.

In Nigeria, HIV transmission among drug users, particularly those who inject drugs (PWIDs), is of significant concern. Shared needles and syringes are a primary driver of HIV transmission among PWIDs. Female sex workers who inject drugs face the highest HIV prevalence risks.

There is no known cure or vaccine for AIDS. However, anti-retroviral therapy (ART) improves the health of the infected individuals and subsequently reduces their risk of death. This involves a combination of drugs, often referred to as “highly active antiretroviral therapy” or HAART. In both high-income and low-income countries, the life expectancy of patients infected with HIV who have access to ART is now measured in decades. It may approach that of the uninfected population in patients who receive optimal treatment [8].

ART treatment still presents substantial limitations, including incomplete health restoration, associated side effects, expensive medications, and a lack of curative [9]. The number of AIDS deaths declined, with around 1.6 million AIDS deaths in 2012, down from 2.3 million in 2005, because of an increase in the use of ART [10]. For prevention, health experts recommend total abstinence from unprotected sex and the use of condoms during casual sex, adequate checks before blood transfusion, and avoidance of shared needles.

With the latest discoveries, health experts have introduced Pre-exposure Prophylaxis (PrEP) to block infection. PrEP is a medicine taken by people who are HIV-negative to reduce the risk of getting HIV. Therapeutic drugs that can prevent individuals who are newly exposed to HIV infection from contracting the disease have also been developed. Post-Exposure Prophylaxis (PEP) blocks HIV infection by preventing the virus from replicating and establishing itself in the body. It does this through a combination of antiretroviral medications that interfere with different stages of the virus’s replication, effectively stopping it from spreading. PEP means taking anti-retroviral drugs, within 72 hours after being potentially exposed to HIV infection, to prevent becoming infected [11,12].

Many authors have modeled on HIV/AIDS transmission. Sharomi *et al*, presented a deterministic HIV treatment model, which incorporates a wild (drug-sensitive) and drug-resistant strain, for gaining insight into the dynamical features of the two strains, and determining effective ways to control HIV spread under this situation. Qualitative analysis of their model reveals that it has a globally asymptotically stable disease-free equilibrium whenever a certain epidemiological threshold $\left({R_{o}}^{t}\right)$ is less than unity, and that the disease will persist in the population when this threshold exceeds unity [13].

Gumel in his article ‘Modeling Transmission Dynamics of HIV/AIDS: Some Results and challenges’ [14], noted that for HIV to be suppressed, Anti retro-viral drugs (ARVs) must be used over a long period of time to reduce the viral load in HIV infected individuals to a non detectable levels, but quickly noted that the use of these ARVs can lead to the emergence and spread of resistant HIV strain. He therefore postulated a targeted (viral-load CD-dependent) treatment. Only HIV infected individuals with low CD4 count (< 200 cells/ml) should be treated. Those infected individuals with such low CD4 count are at the pre-AIDS or AIDS stage with high viral load.

Many other researchers, like Cheneke K. R., presented deterministic models on HIV/AIDS transmission [15], but have all failed to incorporate the recently-exposed Class, which can be subjected to PEP treatments to prevent the infection.

The United States Centers for Disease Control and Prevention, in their exposition on ‘Post-Exposure Prophylaxis (PEP), noted that post-exposure prophylaxis (PEP) is a way of preventing HIV infection after a possible recent exposure [16]. It involves taking ART medications as soon as possible (before 3 days) after a single high-risk event or after being potentially exposed to HIV, to stop HIV from making copies of itself and spreading throughout the body. This is very useful to those who were accidentally exposed to HIV infection, such as health workers and those who are sexually assaulted by HIV infected persons. Studies suggest that PEP reduces the risk of getting HIV by over 80% if it is started before 72 hours of exposure to the virus and must be taken once or twice for 28 days [17].

With these facts, we therefore include the under-3-day HIV Exposed class in our HIV/AIDS transmission model, where the HIV exposed individuals can be prevented from getting infected after due treatments with PEP.

The work was structured as follows: Introduction, model formulation, model analysis, optimal control model and its numerical simulations, cost-effective analysis, findings, and conclusion.

## 2. Model formulation

Model Assumptions

- The population enters into the susceptible class at a constant rate $\Lambda_{h}$.
- The susceptible class becomes infected with HIV through fluid or blood-to-blood contact with the infected individuals.
- The model does not consider HIV infected persons with no clinical symptoms, migrants with HIV, and those born with the virus or transmissions through sharps.
- Health workers, sexually abused persons, and those who are accidentally exposed to the virus may seek PEP treatment. Both the pre-AIDS and AIDS classes may receive antiviral treatment to reduce the viral load.
- Under 3-day HIV-exposed individuals who have judiciously completed their doses of PEP treatment are prevented from getting HIV infection and therefore remain uninfected.
- Only pre-AIDS, those on Treatment, and the AIDS individuals contribute to the viral transmission. The under-3-day Exposed are people who have had less than 72 hours of effective contact with the virus, are not yet infected, and cannot transmit the disease.

We formulate a deterministic model of HIV/AIDS transmission dynamics. The human population is divided into six compartments based on the epidemiological status of individuals. The compartments are;

**Susceptible class** $\left(S_{h}\right)$: these are healthy people, not yet exposed to the virus, and are at risk of being exposed to HIV.
**Under-3-day Exposed class** $\left(E_{h}\right)$: these are individuals who were newly exposed to the virus and are at risk of contracting the disease. The duration for this group is under 72 hours after effective contact with the infected. An under-3-day HIV exposed individual is not yet infected and therefore assumed to be unable to transmit the virus during this period.
**Pre-AIDS class** $\left(I_{h}\right)$: these are individuals who are infected and infectious but have not progressed to the AIDS class. They do not have AIDS symptoms.
**Treatment class** $\left(T_{h}\right)$: these are the under-3-day HIV Exposed, pre-AIDS, and AIDS individuals who are receiving treatment. Some of the under-3-day HIV exposed individuals receive PEP treatment to prevent the infection and progress to the uninfected class.
**Exposed-Uninfected class** $\left(U_{h}\right)$: these are under-3-day HIV exposed individuals who have been satisfactorily free of the virus after receiving PEP treatments.
**AIDS class** $\left(A_{h}\right)$: these are individuals who have the symptoms of AIDS.

The total human population at time t, represented by $N_{h}\left(t\right)$, is obtained as; $N_{h}\left(t\right)\;=\;S_{h}\left(t\right)\;+\;E_{h}\left(t\right)\;+\;I_{h}\left(t\right)\;+\;T_{h}\left(t\right)\;+\;U_{h}\left(t\right)+A_{h}\left(t\right)$

The recruitment rate of humans into the susceptible population is denoted as Λ. Susceptible individuals make sufficient contact with the pre-AIDS, infected individuals on treatment, or the AIDS individuals, to progress to the under-3-day exposed window period. At this stage, the infection can be prevented with PEP treatment. They may opt for treatment or progress to the pre-AIDS class. The period of this under-3-day Exposed class is short-lived (within 72 hours of the contact). This class gets exposed to HIV infection at a variable rate of

$\xi =\frac{\beta \left(\left(1-\varepsilon \right)T_{h}+\eta_{\text{1}}I_{h}+\eta_{\text{2}}A_{h}\right)}{N_{h}},$ known as the force of infection.

β is the effective contact rate and $\eta_{i},\text{\textbackslash{}hspace\{0.33em}\}\left(i=1,2\right),$ represents the increase in relative infectiousness rates of individuals in classes $I_{h}$ and $A_{h}$, compared to those in $T_{h}.$ (1−ε) represents the proportion of those on treatment that can transmit the virus.

Individuals in the pre-AIDS class progress to the AIDS class at the rate l, or seek ART treatment at the rate ρ. The rest of the parameters are as described in Table 1

**Table 1:** Descriptions of the parameter.

Parameters	Descriptions
μ	Natural death rate
δ	Death due to the virus
α	The rate of progression from the under-3-day Exposed class to the pre-AIDS class
k	The rate at which the under-3-day Exposed individuals receive PEP treatments
m	The rate at which those on treatment return to the pre-AIDS class due to relaxation of their treatment or failure of the drugs.
v	The rate at which individuals in the AIDS class on antiretroviral treatment (ART) progress to the treatment class.
ψ	The rate at which those in the under-3-day exposed class who are on PEP treatment progress to the Exposed-Uninfected class.
ε	The fraction of those on treatment who were exposed but uninfected.
ϖ	Fraction of the under-3-day Exposed individuals that receive PEP treatment.

**Fig 1:**
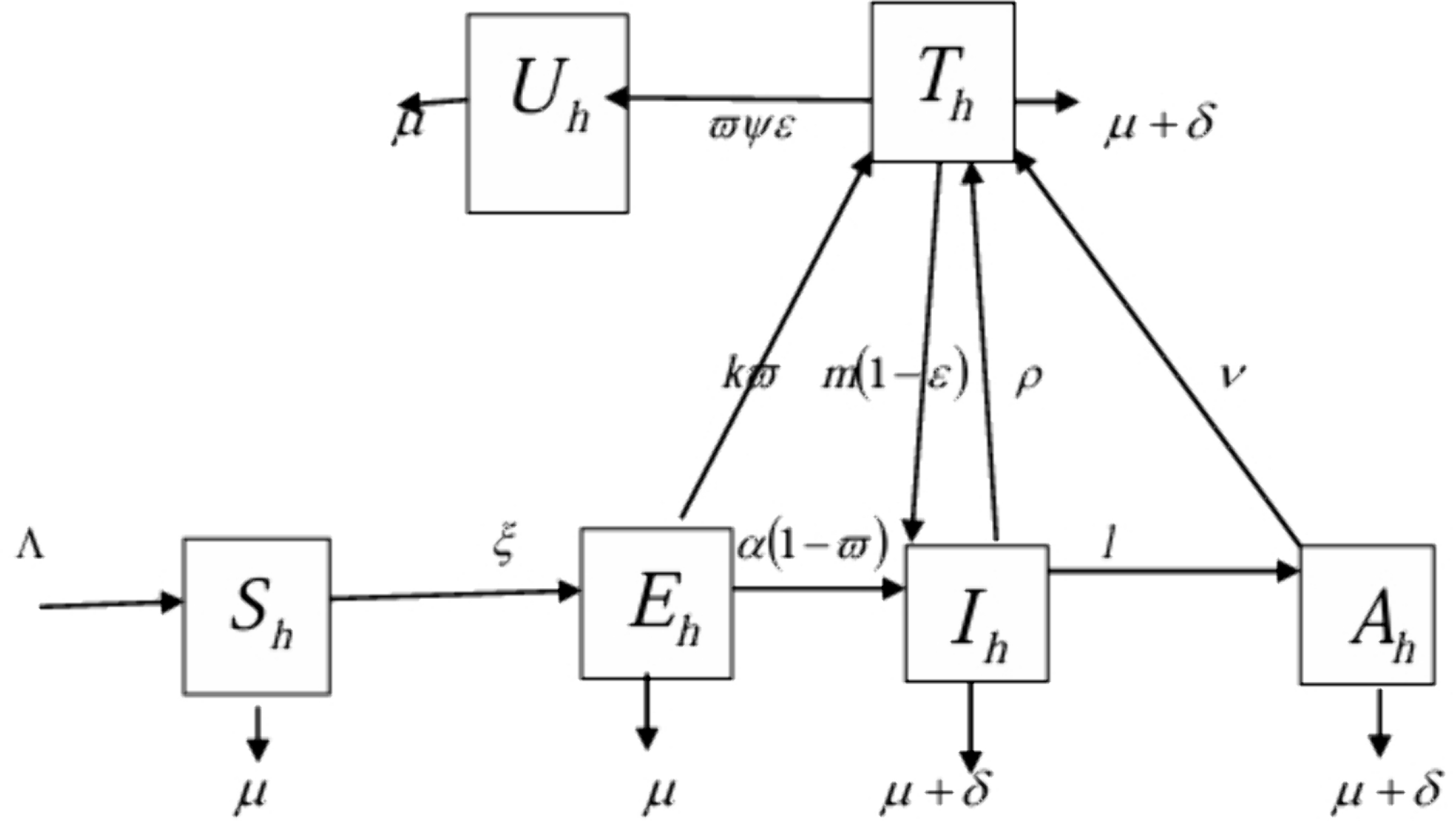
Flow diagram of HIV transmission dynamics.

The transmission dynamics of the virus are represented in the compartmental flow diagram shown in Fig 1.

ϖψε is the rate at which a fraction of the under-3-day exposed individuals who received PEP progress to the Exposed but uninfected class.

Based on the stated assumptions, parameter descriptions, and flow diagram in Fig 1, we present a system of equations governing HIV/AIDS transmission dynamics as (1).


$$\left\{ \begin{array}{c} @l@\frac{dS_{h}}{dt}=\Lambda -\left(\xi +\mu \right)S_{h}\mathrm{\backslash vspace}1mm\\ \frac{dE_{h}}{dt}=\xi S_{h}-\left(k\varpi +\alpha \left(1-\varpi \right)+\mu \right)E_{h}\mathrm{\backslash vspace}1mm\\ \frac{dI_{h}}{dt}=\alpha \left(1-\varpi \right)E_{h}+m\left(1-\varepsilon \right)T_{h}-\left(\rho +l+\mu +\delta \right)I_{h}\mathrm{\backslash vspace}1mm\\ \frac{dT_{h}}{dt}=k\varpi E_{h}+\rho I_{h}+\nu A_{h}-\left(\varpi \psi \varepsilon +m\left(1-\varepsilon \right)+\mu +\delta \right)T_{h}\mathrm{\backslash vspace}1mm\\ \frac{dU_{h}}{dt}=\varpi \psi \varepsilon T_{h}-\left(\mu \right)U_{h}\mathrm{\backslash vspace}1mm\\ \frac{dA_{h}}{dt}=lI_{h}-\left(\nu +\mu +\delta \right)A_{h} \end{array}\right.$$ (1)

With the initial conditions


$$S_{h}\left(0\right)>0,\text{\textbackslash{}hspace\{0.33em}\}E_{h}\left(0\right)>0,\text{\textbackslash{}hspace\{0.33em}\}I_{h}\left(0\right)>0,T_{h}\left(0\right)>0,\text{\textbackslash{}hspace\{0.33em}\}U_{h}\left(0\right)>0,A_{h}\left(0\right)>0$$

## 3. Model analysis

### 3.1 Positivity invariance and boundedness of solutions

Since the system of equations in (1) represents human populations, it is necessary to consider that the associated population sizes cannot be negative. We establish the positive invariant of the epidemiology region Ω given as;


$$\Omega =\left\{\left(S_{h},E_{h},I_{h},T_{h},U_{h},A_{h}\right)\in \overset{\text{6}}{\underset{+}{ℜ}}:N_{h}\leq \frac{\Lambda }{\mu }\right\}$$

**Theorem 1**: All the solutions of the system (1) are positive in the region $\Omega \subset \overset{\text{6}}{\underset{+}{ℜ}}$ for all t>0 provided that the initial conditions are positive.

Proof: Let Ω represent the feasible region of the HIV/AIDS model (1), expressed as $\Omega \subset \overset{\text{6}}{\underset{+}{ℜ}}$ we show the conditions for the positive invariant of Ω

We obtain the rate of change of the total population $\frac{dN}{dt}$ by adding all the equations in (1) to have;


$$\begin{array}{c} \frac{dN}{dt}=\frac{dS_{h}}{dt}+\frac{dE_{h}}{dt}+\frac{dI_{h}}{dt}+\frac{dT_{h}}{dt}+\frac{dU_{h}}{dt}+\frac{dA_{h}}{dt}\\ \Rightarrow \frac{dN}{dt}=\Lambda -\left(S_{h}+E_{h}+I_{h}+T_{h}+U_{h}+A_{h}\right)\mu -\left(I_{h}+T_{h}+A_{h}\right)\delta \\ \Rightarrow \frac{dN}{dt}=\Lambda -N_{h}\mu -\left(I_{h}+T_{h}+A_{h}\right)\delta \\ \Rightarrow \frac{dN}{dt}\leq \Lambda -N_{h}\mu \end{array}$$

$$\frac{dN}{dt}+N_{h}\mu \leq \Lambda$$

Multiplying through with the integrating factor $e^{\int \mu dt}=e^{\mu t}$, and integrating gives;


$$\begin{array}{c} Ne^{\mu t}\leq \int \Lambda e^{\mu t}\\ Ne^{\mu t}\leq \frac{\Lambda e^{\mu t}}{\mu }+c \end{array}$$

Solving for the constant c, we apply the initial conditions, when t=0, and substitute back to have;

$N\left(t\right)\leq \frac{\Lambda }{\mu }+\left(N\left(0\right)-\frac{\Lambda }{\mu }\right)e^{-\mu t}.$ As t→∞, we have $N\left(t\right)\leq \frac{\Lambda }{\mu }$

For the initial population $0<N\left(0\right)\leq \frac{\Lambda }{\mu }$, then $0<N\left(t\right)\leq \frac{\Lambda }{\mu }$ for all t>0. This implies that the model system (1) is positive invariance and that for all t>0, every solution of the model system (1) with the initial condition in the region Ω remains there and is all bounded.

### 3.2 Disease-free equilibrium state $\left(\varepsilon_{\text{0}}\right)$

The HIV/AIDS disease-free equilibrium (DFE) state implies a state where the disease is absent in the population. The DFE is obtained by setting the right-hand side of Equations (1) to zero in the absence of HIV infection. That is;


$$\frac{dS_{h}}{dt}=\frac{dE_{h}}{dt}=\frac{dI_{h}}{dt}=\frac{dT_{h}}{dt}=\frac{dU_{h}}{dt}=\frac{dA_{h}}{dt}=0$$

In the absence of the disease, we have $E_{h}=I_{h}=T_{h}=U_{h}=A_{h}=0$. This leads Equations (1) to;

$\frac{dS_{h}}{dt}=\Lambda -\left(\xi +\mu \right)S_{h}=0$. The force of infection ξ is also zero at the DFE state, which implies that $S_{h}=\frac{\Lambda }{\mu }$. Thus, the DFE point is $\varepsilon_{\text{0}}=\left(\frac{\Lambda }{\mu },0,0,0,0,0\right)$

### 3.3 Effective reproduction number, $R_{e}.$

This implies the mean number of new infections generated by a typical infectious individual introduced in a population [18,19]. It is a threshold quantity to determine the system’s stability. To compute the reproduction number $\left(R_{e}\right)$, we employ the method of the next generation matrix as in [14,20]. $R_{e}$ is the spectral radius of the next generation matrix. In this approach, we identify the new infection terms F and the remaining transition terms V, of the infection. The classes where HIV is present are $E_{h},I_{h},T_{h}$ and $A_{h}$. We can then write (1) as X=F×V and$V=V^{-}-V^{+}$, where $X=\left(S_{h},E_{h},I_{h},T_{h},U_{h},A_{h}\right)$, $V^{-}$is the rate of transfer of the infectious individuals out of each class, and $V^{+}$ is the rate of transfer into each class by all other means. Hence, the associated matrices F of the new infection terms and V, the remaining transition terms are given by.


$$F=\left[\begin{array}{c} @c@\frac{\beta \left(\left(1-\varepsilon \right)T_{h}+\eta_{\text{1}}I_{h}+\eta_{\text{2}}A_{h}\right)}{N_{h}}S_{h}\\ 0\\ 0\\ 0 \end{array}\right]$$

and


$$V=\left[\begin{array}{c} @c@\left(k\varpi +\alpha \left(1-\varpi \right)+\mu \right)E_{h}\\ \left(\rho +l+\mu +\delta \right)I_{h}-\alpha \left(1-\varpi \right)E_{h}-m\left(1-\varepsilon \right)T_{h}\\ \left(\varpi \psi \varepsilon +m\left(1-\varepsilon \right)+\mu +\delta \right)T_{h}-k\varpi E_{h}-\rho I_{h}-\nu A_{h}\\ \left(\nu +\mu +\delta \right)A_{h}-lI_{h} \end{array}\right]\;,$$ (2)

We obtain the Jacobian matrices of F and V with respect to the disease-free equilibrium $\varepsilon_{\text{0}}=\left(\frac{\Lambda }{\mu },0,0,0,0,0\right).$ Let the Jacobian matrices of F and V be f and v respectively. Then,

$f=\left[\begin{array}{cccc} @cccc@0 & \frac{\beta \eta_{\text{1}}S_{h}}{N_{h}} & \frac{\beta \left(1-\varepsilon \right)S_{h}}{N_{h}} & \frac{\beta \eta_{\text{2}}S_{h}}{N_{h}}\\ 0 & 0 & 0 & 0\\ 0 & 0 & 0 & 0\\ 0 & 0 & 0 & 0 \end{array}\right]$ and


$$\begin{array}{c} v=\left[\begin{array}{cccc} @cccc@\left(k\varpi +\alpha \left(1-\varpi \right)+\mu \right) & 0 & 0 & 0\\ -\alpha \left(1-\varpi \right) & \left(\rho +l+\mu +\delta \right) & -m\left(1-\varepsilon \right) & 0\\ -k\varpi & -\rho & \left(\varpi \psi \varepsilon +m\left(1-\varepsilon \right)+\mu +\delta \right) & -\nu \\ 0 & -l & 0 & \left(\nu +\mu +\delta \right) \end{array}\right] \end{array}$$ (3)

For computation, let

$f=\left[\begin{array}{cccc} @cccc@0 & A & B & C\\ 0 & 0 & 0 & 0\\ 0 & 0 & 0 & 0\\ 0 & 0 & 0 & 0 \end{array}\right]$ and $v=\left[\begin{array}{cccc} @cccc@W & 0 & 0 & 0\\ -g & X & -h & 0\\ -i & -j & Y & -k\\ 0 & -l & 0 & Z \end{array}\right]$

Computing with Matlab gives the product of f and the inverse of v as the matrix,


$$\begin{array}{c} fv^{-1}=\left[\begin{array}{cccc} @cccc@\frac{-AZ\left(gY+ih\right)-B\left(gjZ+gkl+iXZ\right)-Cl\left(gY+ih\right)}{\left(-XYZ+jhZ+lkh\right)} & \frac{-AYZ-B\left(jZ+kl+iXZ\right)-CYl}{\left(-XYZ+jhZ+lkh\right)} & \frac{-AhZ-BXZ-Chl}{\left(-XYZ+jhZ+lkh\right)} & \frac{-Ahk-BXk-C\left(-XY+hj\right)}{\left(-XYZ+jhZ+lkh\right)}\\ 0 & 0 & 0 & 0\\ 0 & 0 & 0 & 0\\ 0 & 0 & 0 & 0 \end{array}\right] \end{array}$$

For the eigenvalues $\lambda_{i},\text{\textbackslash{}hspace\{1em}\}i=1,2,3,4$, we solve for λ in the characteristic equation $|fv^{-1}-I\lambda |=0$,


$$\begin{array}{c} \left|\begin{array}{cccc} \frac{-AZ\left(gY+ih\right)-B\left(gjZ+gkl+iXZ\right)-Cl\left(gY+ih\right)}{W\left(-XYZ+jhZ+lkh\right)}-\lambda & \frac{-AYZ-B\left(jZ+kl+iXZ\right)-CYl}{\left(-XYZ+jhZ+lkh\right)} & \frac{-AhZ-BXZ-Chl}{\left(-XYZ+jhZ+lkh\right)} & \frac{-Ahk-BYk-C\left(-XY+hj\right)}{\left(-XYZ+jhZ+lkh\right)}\\ 0 & -\lambda & 0 & 0\\ 0 & 0 & -\lambda & 0\\ 0 & 0 & 0 & -\lambda \end{array}\right|=0 \end{array}$$

to have

$\lambda_{\text{1}}=\frac{AZ\left(gY+ih\right)+B\left(gjZ+gkl+iXZ\right)+Cl\left(gY+ih\right)}{W\left(XYZ-jhZ-lkh\right)}$, $\lambda_{\text{2}}=0,$ $\lambda_{\text{3}}=0,$ and $\lambda_{\text{4}}=0,$

Substituting back the parameters in $\lambda_{\text{1}},$to have


$$\lambda_{\text{1}}=\frac{\frac{\beta \eta_{\text{1}}S_{h}}{N_{h}}Z\left(a\left(1-\omega \right)Y+k\omega m\left(1-\varepsilon \right)\right)+\frac{\beta \left(1-\varepsilon \right)S_{h}}{N_{h}}\left(a\left(1-\omega \right)\left(\rho Z+vl\right)+kwXZ\right)+\frac{\beta \eta_{\text{2}}S_{h}}{N_{h}}l\left(\alpha \left(1-\omega \right)Y+k\omega m\left(1-\varepsilon \right)\right)}{W\left(XYZ-\rho m\left(1-\varepsilon \right)Z-lvm\left(1-\varepsilon \right)\right)}$$ (4)

where, W=(kϖ+α(1−ϖ)+μ),\hspace{0.33em}X=(ρ+l+μ+δ),\hspace{0.33em}Y=(ϖψε+m(1−ε)+μ+δ)\hspace{0.33em}and\hspace{0.33em}Z==(ν+μ+δ) At DFE, $S_{h}=\frac{\Lambda }{\mu }$ and the dominant eigenvalue (the spectral radius of the matrix) is $\lambda_{\text{1}}$. Hence, the effective reproduction number is given as;


$$R_{e}=\frac{\beta \Lambda }{\mu N_{h}}\left(\frac{\eta_{\text{1}}Z\left(\alpha \left(1-\omega \right)Y+k\omega m\left(1-\varepsilon \right)\right)+\left(1-\varepsilon \right)\left(\alpha \left(1-\omega \right)\left(\rho Z+vl\right)+kwXZ\right)+\eta_{\text{2}}l\left(\alpha \left(1-\omega \right)Y+k\omega m\left(1-\varepsilon \right)\right)}{W\left(XYZ-\rho m\left(1-\varepsilon \right)Z-lvm\left(1-\varepsilon \right)\right)}\right)$$ (5)

To show that $\lambda_{\text{1}}>0$, we simplify the denominator of (5) to clear the negative signs;

That is W(XYZ−ρm(1−ε)Z−lvm(1−ε))=W(Z(XY−ρm(1−ε))−lvm(1−ε))

Substitute the values of XY and expand to have $\begin{array}{c} W\left(Z\left(\left(\rho +\left(l+\mu +\delta \right)\right)\left(\varpi \psi \varepsilon +m\left(1-\varepsilon \right)+\mu +\delta \right)-\rho m\left(1-\varepsilon \right)\right)-lvm\left(1-\varepsilon \right)\right)\\ =W\left(Z\rho \left(\psi \varepsilon +\mu +\delta \right)+Z\left(l+\mu +\delta \right)\left(\varpi \psi \varepsilon +\mu +\delta \right)+Zm\left(1-\varepsilon \right)\left(l+\mu +\delta \right)-lvm\left(1-\varepsilon \right)\right) \end{array}$

Substitute the value of Z in the third term and simplify to have;


$$\begin{array}{c} W\left(\begin{array}{c} @l@Z\rho \left(\varpi \psi \varepsilon +\mu +\delta \right)+Z\left(l+\mu +\delta \right)\left(\varpi \psi \varepsilon +\mu +\delta \right)+vm\left(1-\varepsilon \right)l+vm\left(1-\varepsilon \right)\left(\mu +\delta \right)\\ +\left(\mu +\delta \right)\left(m\left(1-\varepsilon \right)l\right)+{\left(\mu +\delta \right)}^{\text{2}}m\left(1-\varepsilon \right)-lvm\left(1-\varepsilon \right) \end{array}\right)\\ =W\left(Z\left(\varpi \psi \varepsilon +\mu +\delta \right)\left(\rho +l+\mu +\delta \right)+m\left(1-\varepsilon \right)\left(\mu +\delta \right)\left(v+l+\mu +\delta \right)\right)\\ =W\left(Z\left(\varpi \psi \varepsilon +\mu +\delta \right)X+m\left(1-\varepsilon \right)\left(\mu +\delta \right)\left(l+Z\right)\right) \end{array}$$

Giving; $\begin{array}{c} \lambda_{\text{1}}=\frac{\frac{\beta \eta_{\text{1}}S_{h}}{N_{h}}Z\left(\alpha \left(1-\omega \right)Y+k\omega m\left(1-\varepsilon \right)\right)+\frac{\beta \left(1-\varepsilon \right)S_{h}}{N_{h}}\left(\alpha \left(1-\omega \right)\left(\rho Z+vl\right)+kwXZ\right)+\frac{\beta \eta_{\text{2}}S_{h}}{N_{h}}l\left(\alpha \left(1-\omega \right)Y+k\omega m\left(1-\varepsilon \right)\right)}{W\left(Z\left(\varpi \psi \varepsilon +\mu +\delta \right)X+m\left(1-\varepsilon \right)\left(\mu +\delta \right)\left(l+Z\right)\right)}\\ \text{\textbackslash{}hspace\{1em}\}=\frac{\beta S_{h}}{N_{h}}\left(\frac{\eta_{\text{1}}Z\left(\alpha \left(1-\omega \right)Y+k\omega m\left(1-\varepsilon \right)\right)+\left(1-\varepsilon \right)\left(\alpha \left(1-\omega \right)\left(\rho Z+vl\right)+kwXZ\right)+\eta_{\text{2}}l\left(\alpha \left(1-\omega \right)Y+k\omega m\left(1-\varepsilon \right)\right)}{W\left(Z\left(\varpi \psi \varepsilon +\mu +\delta \right)X+m\left(1-\varepsilon \right)\left(\mu +\delta \right)\left(l+Z\right)\right)}\right)\\ for\text{\textbackslash{}hspace\{0.33em}\}0<\varepsilon ,\varpi <1 \end{array}$ Thus,


$$R_{e}=\frac{\beta \Lambda }{\mu N_{h}}\left(\frac{\eta_{\text{1}}Z\left(\alpha \left(1-\omega \right)Y+k\omega m\left(1-\varepsilon \right)\right)+\left(1-\varepsilon \right)\left(\alpha \left(1-\omega \right)\left(\rho Z+vl\right)+kwXZ\right)+\eta_{\text{2}}l\left(\alpha \left(1-\omega \right)Y+k\omega m\left(1-\varepsilon \right)\right)}{W\left(Z\left(\varpi \psi \varepsilon +\mu +\delta \right)X+m\left(1-\varepsilon \right)\left(\mu +\delta \right)\left(l+Z\right)\right)}\right)$$ (6)

### 3.4 Local stability of DFE

**Theorem 2**: The Disease Free Equilibrium point is said to be locally asymptotically stable if the effective reproduction number is less than unity $\left(R_{e}<1\right)$ and unstable if $R_{e}>1$.

Proof:

We obtained the Jacobian matrix of the system (1) at the DFE $\left(\varepsilon_{\text{0}}\right)$,


$$\begin{array}{c} J\left(\varepsilon_{\text{0}}\right)=\left[\begin{array}{cccccc} @cccccc@-\mu & 0 & -\frac{\beta \eta_{\text{1}}\Lambda }{N_{h}\mu } & -\frac{\beta \left(1-\varepsilon \right)\Lambda }{N_{h}\mu } & 0 & -\frac{\beta \eta_{\text{2}}\Lambda }{N_{h}\mu }\\ 0 & -\left(k\varpi +a\left(1-\varpi \right)+\mu \right) & \frac{\beta \eta_{\text{1}}\Lambda }{N_{h}\mu } & \frac{\beta \left(1-\varepsilon \right)\Lambda }{N_{h}\mu } & 0 & \frac{\beta \eta_{\text{2}}\Lambda }{N_{h}\mu }\\ 0 & a\left(1-\varpi \right) & -\left(\rho +l+\mu +\delta \right) & m\left(1-\varepsilon \right) & 0 & 0\\ 0 & k\varpi & \rho & -\left(\varpi \psi \varepsilon +m\left(1-\varepsilon \right)+\mu +\delta \right) & 0 & v\\ 0 & 0 & 0 & \varpi \psi \varepsilon & -\left(\mu \right) & 0\\ 0 & 0 & l & 0 & 0 & -\left(v+\mu +\delta \right) \end{array}\right] \end{array}$$

Letting W=(kϖ+a(1−ϖ)+μ),\hspace{0.33em}X=(ρ+l+μ+δ),\hspace{0.33em}Y=(ϖψε+m(1−ε)+μ+δ),\hspace{0.33em}and\hspace{0.33em}Z=(v+μ+δ), the eigenvalues of the Jacobian matrix are given as;


$$\begin{array}{c} \left|\left[\begin{array}{cccccc} @cccccc@-\mu -\lambda & 0 & -\frac{\beta \eta_{\text{1}}\Lambda }{N_{h}\mu } & -\frac{\beta \left(1-\varepsilon \right)\Lambda }{N_{h}\mu } & 0 & -\frac{\beta \eta_{\text{2}}\Lambda }{N_{h}\mu }\\ 0 & -W-\lambda & \frac{\beta \eta_{\text{1}}\Lambda }{N_{h}\mu } & \frac{\beta \left(1-\varepsilon \right)\Lambda }{N_{h}\mu } & 0 & \frac{\beta \eta_{\text{2}}\Lambda }{N_{h}\mu }\\ 0 & a\left(1-\varpi \right) & -X-\lambda & m\left(1-\varepsilon \right) & 0 & 0\\ 0 & k\varpi & \rho & -Y-\lambda & 0 & v\\ 0 & 0 & 0 & \varpi \psi \varepsilon & -\left(\mu \right)-\lambda & 0\\ 0 & 0 & l & 0 & 0 & -Z-\lambda \end{array}\right]\right|=0 \end{array}$$

$$\begin{array}{c} \Rightarrow \left(-\mu -\lambda \right)\left(-\mu -\lambda \right)\left|\left[\begin{array}{cccc} @cccc@-W-\lambda & \frac{\beta \eta_{\text{1}}\Lambda }{N_{h}\mu } & \frac{\beta \left(1-\varepsilon \right)\Lambda }{N_{h}\mu } & \frac{\beta \eta_{\text{2}}\Lambda }{N_{h}\mu }\\ a\left(1-\varpi \right) & -X-\lambda & m\left(1-\varepsilon \right) & 0\\ k\varpi & \rho & -Y-\lambda & v\\ 0 & l & 0 & -Z-\lambda \end{array}\right]\right|=0 \end{array}$$

This implies that

(−μ−λ)(−μ−λ)=0 or $\left|\left[\begin{array}{cccc} @cccc@-W-\lambda & \frac{\beta \eta_{\text{1}}\Lambda }{N_{h}\mu } & \frac{\beta \left(1-\varepsilon \right)\Lambda }{N_{h}\mu } & \frac{\beta \eta_{\text{2}}\Lambda }{N_{h}\mu }\\ a\left(1-\varpi \right) & -X-\lambda & m\left(1-\varepsilon \right) & 0\\ k\varpi & \rho & -Y-\lambda & v\\ 0 & l & 0 & -Z-\lambda \end{array}\right]\right|=0$

But $\left(-\mu -\lambda \right)\left(-\mu -\lambda \right)=\lambda^{\text{2}}+2\lambda \left(\mu \right)+\left(\mu^{\text{2}}\right)\neq 0$

Since \hspace{0.33em}μ is a positive number.

Hence,$\begin{array}{c} \left[\begin{array}{cccc} @cccc@-W-\lambda & \frac{\beta \eta_{\text{1}}\Lambda }{N_{h}\mu } & \frac{\beta \left(1-\varepsilon \right)\Lambda }{N_{h}\mu } & \frac{\beta \eta_{\text{2}}\Lambda }{N_{h}\mu }\\ a\left(1-\varpi \right) & -X-\lambda & m\left(1-\varepsilon \right) & 0\\ k\varpi & \rho & -Y-\lambda & v\\ 0 & l & 0 & -Z-\lambda \end{array}\right]=0\\ \Rightarrow -\left(W+\lambda \right)\left|\begin{array}{ccc} -X-\lambda & m\left(1-\varepsilon \right) & 0\\ \rho & -Y-\lambda & v\\ l & 0 & -Z-\lambda \end{array}\right|-a\left(1-\varpi \right)\left|\begin{array}{ccc} \frac{\beta \eta_{\text{1}}\Lambda }{N_{h}\mu } & \frac{\beta \left(1-\varepsilon \right)\Lambda }{N_{h}\mu } & \frac{\beta \eta_{\text{2}}\Lambda }{N_{h}\mu }\\ \rho & -Y-\lambda & v\\ l & 0 & -Z-\lambda \end{array}\right|+k\varpi \left|\begin{array}{ccc} \frac{\beta \eta_{\text{1}}\Lambda }{N_{h}\mu } & \frac{\beta \left(1-\varepsilon \right)\Lambda }{N_{h}\mu } & \frac{\beta \eta_{\text{2}}\Lambda }{N_{h}\mu }\\ -X-\lambda & m\left(1-\varepsilon \right) & 0\\ l & 0 & -Z-\lambda \end{array}\right|=0 \end{array}$ after which the expansion and simplifications give the following polynomial;


$$\begin{array}{c} \lambda^{\text{4}}+\lambda^{\text{3}}\left(W+X+Y+Z\right)+\lambda^{\text{2}}\left(WZ+WX-\frac{kw\beta \left(1-\varepsilon \right)\Lambda }{N_{h}\mu }+XY+XZ+YZ-\rho m\left(1-\varepsilon \right)+WY-\frac{a\left(1-\varpi \right)\beta \eta_{\text{1}}\Lambda }{N_{h}\mu }\right)\\ +\lambda \left(\begin{array}{c} @l@\frac{a\left(1-\varpi \right)\rho \beta \left(1-\varepsilon \right)\Lambda }{N_{h}\mu }-\frac{a\left(1-\varpi \right)l\beta \eta_{\text{2}}\Lambda }{N_{h}\mu }-\frac{kw\beta \eta_{\text{1}}\Lambda m\left(1-\varepsilon \right)}{N_{h}\mu }-\frac{a\left(1-\varpi \right)\beta \eta_{\text{1}}\Lambda Y}{N_{h}\mu }-\frac{a\left(1-\varpi \right)\beta \eta_{\text{1}}\Lambda Z}{N_{h}\mu }\\ +WXY+WXZ+WYZ-\frac{k\varpi \beta \left(1-\varepsilon \right)\Lambda X}{N_{h}\mu }+XYZ-\rho m\left(1-\varepsilon \right)Z-lm\left(1-\varepsilon \right)v-W\rho m\left(1-\varepsilon \right)-\frac{k\varpi \beta \left(1-\varepsilon \right)\Lambda Z}{N_{h}\mu } \end{array}\right)\\ +WXYZ-W\rho m\left(1-\varepsilon \right)Z-Wlm\left(1-\varepsilon \right)v-\frac{a\left(1-\varpi \right)\beta \eta_{\text{1}}\Lambda YZ}{N_{h}\mu }-\frac{a\left(1-\varpi \right)\rho \beta \left(1-\varepsilon \right)\Lambda Z}{N_{h}\mu }-\frac{a\left(1-\varpi \right)l\beta \left(1-\varepsilon \right)\Lambda v}{N_{h}\mu }\\ -\frac{a\left(1-\varpi \right)l\beta \eta_{\text{2}}\Lambda Y}{N_{h}\mu }-\frac{k\varpi \beta \eta_{\text{1}}\Lambda m\left(1-\varepsilon \right)Z}{N_{h}\mu }-\frac{k\varpi X\beta \left(1-\varepsilon \right)\Lambda Z}{N_{h}\mu }-\frac{k\varpi l\beta \eta_{\text{2}}\Lambda m\left(1-\varepsilon \right)}{N_{h}\mu } \end{array}$$

$$\begin{array}{c} \Rightarrow \lambda^{\text{4}}+\lambda^{\text{3}}\left(W+X+Y+Z\right)+\lambda^{\text{2}}\left(WZ+WX+XY+XZ+YZ+WY-\frac{\beta \Lambda }{N_{h}\mu }\left(kw\left(1-\varepsilon \right)+a\left(1-\varpi \right)\eta_{\text{1}}\right)-\rho m\left(1-\varepsilon \right)\right)\\ +\lambda \left(\begin{array}{c} @l@WXY+WXZ+WYZ+XYZ+\frac{\beta \Lambda }{N_{h}\mu }\left(a\left(1-\varpi \right)\left(\rho \left(1-\varepsilon \right)-l\eta_{\text{2}}-\eta_{\text{1}}Y-\eta_{\text{1}}Z\right)-k\varpi \left(1-\varepsilon \right)\left(\eta_{\text{1}}m+X+Z\right)\right)\\ -m\left(1-\varepsilon \right)\left(\rho Z-lv-W\rho \right) \end{array}\right)\\ +WXYZ-W\rho m\left(1-\varepsilon \right)Z-Wlm\left(1-\varepsilon \right)v-\frac{\beta \Lambda }{N_{h}\mu }\left(\begin{array}{c} @l@a\left(1-\varpi \right)\left(\eta_{\text{1}}YZ+\rho \left(1-\varepsilon \right)Z+l\left(1-\varepsilon \right)v+l\eta_{\text{2}}Y\right)\\ +\left(1-\varepsilon \right)\left(mk\varpi \eta_{\text{1}}Z+k\varpi X\left(1-\varepsilon \right)Z+k\varpi ml\eta_{\text{2}}\right) \end{array}\right)=0 \end{array}$$ (7)

This can be expressed as $\lambda^{\text{4}}A_{\text{0}}+\lambda^{\text{3}}A_{\text{1}}+\lambda^{\text{2}}A_{\text{2}}+\lambda A_{\text{3}}+A_{\text{4}}=0$, where;

$A_{\text{0}}=1,$ $A_{\text{1}}=W+X+Y+Z$ $A_{\text{2}}=WZ+WX+XY+XZ+YZ+WY-\frac{\beta \Lambda }{N_{h}\mu }\left(kw\left(1-\varepsilon \right)+a\left(1-\varpi \right)\eta_{\text{1}}\right)-\rho m\left(1-\varepsilon \right),$ $\begin{array}{c} A_{\text{3}}=WXY+WXZ+WYZ+XYZ+\frac{\beta \Lambda }{N_{h}\mu }\left(a\left(1-\varpi \right)\left(\rho \left(1-\varepsilon \right)-l\eta_{\text{2}}-\eta_{\text{1}}Y-\eta_{\text{1}}Z\right)-k\varpi \left(1-\varepsilon \right)\left(\eta_{\text{1}}m+X+Z\right)\right)\\ \text{\textbackslash{}hspace\{0.33em}\;\;\;\}-m\left(1-\varepsilon \right)\left(\rho Z-lv-W\rho \right), \end{array}$ $A_{\text{4}}=WXYZ-W\rho m\left(1-\varepsilon \right)Z-Wlm\left(1-\varepsilon \right)v-\frac{\beta \Lambda }{N_{h}\mu }\left(\begin{array}{c} @l@a\left(1-\varpi \right)\left(\eta_{\text{1}}YZ+\rho \left(1-\varepsilon \right)Z+l\left(1-\varepsilon \right)v+l\eta_{\text{2}}Y\right)\\ +\left(1-\varepsilon \right)\left(mk\varpi \eta_{\text{1}}Z+k\varpi X\left(1-\varepsilon \right)Z+mk\varpi l\eta_{\text{2}}\right) \end{array}\right)$

By the Routh-Hurwitz criteria, the fourth-order polynomial equation as presented in (7) has strictly negative real part roots, if$\Delta_{\text{0}}=A_{\text{0}}>0,\Delta_{\text{1}}=A_{\text{1}}>0,\text{\textbackslash{}hspace\{0.33em}\}\Delta_{\text{2}}=\left|\begin{array}{cc} A_{\text{1}} & A_{\text{0}}\\ A_{\text{3}} & A_{\text{2}} \end{array}\right|=A_{\text{1}}A_{\text{2}}-A_{\text{3}}A_{\text{0}}>0$ $\Delta_{\text{3}}=\left|\begin{array}{ccc} A_{\text{1}} & A_{\text{0}} & 0\\ A_{\text{3}} & A_{\text{2}} & A_{\text{1}}\\ 0 & A_{\text{4}} & A_{\text{3}} \end{array}\right|=A_{\text{1}}A_{\text{2}}A_{\text{3}}-{A_{\text{1}}}^{\text{2}}A_{\text{4}}-A_{\text{0}}{A_{\text{3}}}^{\text{2}}>0,\text{\textbackslash{}hspace\{0.33em}\}\Delta_{\text{4}}>0$

Otherwise $\varepsilon_{\text{0}}$ is unstable.

It is already established that $\Delta_{\text{0}}=1,\text{\textbackslash{}hspace\{0.33em}\}\Delta_{\text{1}}>0$ since all the parameters are positive.


$$\begin{array}{c} \Delta_{\text{2}}=A_{\text{1}}A_{\text{2}}-A_{\text{3}}A_{\text{0}}>0\\ \Rightarrow \left(W+X+Y+Z\right)\left(WZ+WX+XY+XZ+YZ+WY-\frac{\beta \Lambda }{N_{h}\mu }\left(kw\left(1-\varepsilon \right)+a\left(1-\varpi \right)\eta_{\text{1}}\right)-\rho m\left(1-\varepsilon \right)\right)\\ -\left(\begin{array}{c} @l@WXY+WXZ+WYZ+XYZ+\frac{\beta \Lambda }{N_{h}\mu }\left(a\left(1-\varpi \right)\left(\rho \left(1-\varepsilon \right)-l\eta_{\text{2}}-\eta_{\text{1}}Y-\eta_{\text{1}}Z\right)-k\varpi \left(1-\varepsilon \right)\left(\eta_{\text{1}}m+X+Z\right)\right)\\ \text{\textbackslash{}hspace\{0.33em}\;\;\;\}-m\left(1-\varepsilon \right)\left(\rho Z-lv-W\rho \right), \end{array}\right)>0 \end{array}$$

$$\begin{array}{c} \Rightarrow \left(W+X+Y+Z\right)\left(WZ+WX+XY+XZ+YZ+WY-\frac{\beta \Lambda }{N_{h}\mu }\left(kw\left(1-\varepsilon \right)+a\left(1-\varpi \right)\eta_{\text{1}}\right)-\rho m\left(1-\varepsilon \right)\right)\\ >\left(\begin{array}{c} @l@WXY+WXZ+WYZ+XYZ+\frac{\beta \Lambda }{N_{h}\mu }\left(a\left(1-\varpi \right)\left(\rho \left(1-\varepsilon \right)-l\eta_{\text{2}}-\eta_{\text{1}}Y-\eta_{\text{1}}Z\right)-k\varpi \left(1-\varepsilon \right)\left(\eta_{\text{1}}m+X+Z\right)\right)\\ \text{\textbackslash{}hspace\{0.33em}\;\;\;\}-m\left(1-\varepsilon \right)\left(\rho Z-lv-W\rho \right), \end{array}\right) \end{array}$$

$$\begin{array}{c} \Delta_{\text{3}}=A_{\text{1}}A_{\text{2}}A_{\text{3}}-{A_{\text{1}}}^{\text{2}}A_{\text{4}}-A_{\text{0}}{A_{\text{3}}}^{\text{2}}>0,\\ \Rightarrow \Delta_{\text{3}}=A_{\text{1}}A_{\text{2}}A_{\text{3}}>{A_{\text{1}}}^{\text{2}}A_{\text{4}}+A_{\text{0}}{A_{\text{3}}}^{\text{2}} \end{array}$$

Also,


$$\begin{array}{c} \text{\textbackslash{}hspace\{0.33em}\}\Delta_{\text{4}}>0\\ \Rightarrow WXYZ-W\rho m\left(1-\varepsilon \right)Z-Wlm\left(1-\varepsilon \right)v-\frac{a\left(1-\varpi \right)\beta \eta_{\text{1}}\Lambda YZ}{N_{h}\mu }-\frac{a\left(1-\varpi \right)\rho \beta \left(1-\varepsilon \right)\Lambda Z}{N_{h}\mu }-\frac{a\left(1-\varpi \right)l\beta \left(1-\varepsilon \right)\Lambda v}{N_{h}\mu }\\ -\frac{a\left(1-\varpi \right)l\beta \eta_{\text{2}}\Lambda Y}{N_{h}\mu }-\frac{k\varpi \beta \eta_{\text{1}}\Lambda m\left(1-\varepsilon \right)Z}{N_{h}\mu }-\frac{k\varpi X\beta \left(1-\varepsilon \right)\Lambda m\left(1-\varepsilon \right)Z}{N_{h}\mu }-\frac{k\varpi l\beta \eta_{\text{2}}\Lambda m\left(1-\varepsilon \right)}{N_{h}\mu }>0 \end{array}$$

$$\Rightarrow W\left(XYZ-\rho m\left(1-\varepsilon \right)Z-lm\left(1-\varepsilon \right)v\right)-\frac{\beta \Lambda }{N_{h}\mu }\left(\begin{array}{c} @l@a\left(1-\varpi \right)\eta_{\text{1}}YZ+a\left(1-\varpi \right)\rho \left(1-\varepsilon \right)Z+a\left(1-\varpi \right)l\left(1-\varepsilon \right)v+a\left(1-\varpi \right)l\eta_{\text{2}}Y\\ +k\varpi \eta_{\text{1}}m\left(1-\varepsilon \right)Z+k\varpi X\left(1-\varepsilon \right)Z+k\varpi l\eta_{\text{2}}m\left(1-\varepsilon \right) \end{array}\right)>0$$

$$\begin{array}{c} \Rightarrow W\left(XYZ-\rho m\left(1-\varepsilon \right)Z-lm\left(1-\varepsilon \right)v\right)\\ >\frac{\beta \Lambda }{N_{h}\mu }\left(\eta_{\text{1}}Z\left(a\left(1-\varpi \right)Y+k\varpi m\left(1-\varepsilon \right)\right)+\left(1-\varepsilon \right)\left(\left(a\left(1-\varpi \right)\left(\rho Z+lv\right)+k\varpi XZ\right)\right)+l\eta_{\text{2}}\left(a\left(1-\varpi \right)Y+k\varpi m\left(1-\varepsilon \right)\right)\right) \end{array}$$

$$\begin{array}{c} \Rightarrow \frac{\beta \Lambda }{\mu N_{h}}\left(\frac{\eta_{\text{1}}Z\left(\alpha \left(1-\omega \right)Y+k\omega m\left(1-\varepsilon \right)\right)+\left(1-\varepsilon \right)\left(\alpha \left(1-\omega \right)\left(\rho Z+vl\right)+kwXZ\right)+\eta_{\text{2}}l\left(\alpha \left(1-\omega \right)Y+k\omega m\left(1-\varepsilon \right)\right)}{W\left(XYZ-\rho m\left(1-\varepsilon \right)Z-lvm\left(1-\varepsilon \right)\right)}\right)<1\\ \Rightarrow R_{e}<1 \end{array}$$

Thus, the DFE of system (1) is locally asymptotically stable if $R_{e}<1$. This shows that HIV can be eliminated from the community. This also implies that the initial population sizes in the model are in the basin of attraction of $\varepsilon_{\text{0}}$.

### 3.5 Endemic equilibrium

The endemic equilibrium state is calculated by equating each of the equations of (1) to zero and solving simultaneously for each of the state variables. This is a state where HIV/AIDS is endemic in the population, which we shall denote as $\varepsilon_{h}=\left({S_{h}}^{*},{E_{h}}^{*},{I_{h}}^{*},{T_{h}}^{*},{U_{h}}^{*},{A_{h}}^{*}\right)$


$$\begin{array}{c} \frac{d{S_{h}}^{*}}{dt}=\Lambda -\left(\xi^{*}+\mu \right){S_{h}}^{*}=0\text{\textbackslash{}hspace\{1em}\;\;\;\;\;\}\mathrm{\backslash vspace}1mm\\ \frac{d{E_{h}}^{*}}{dt}=\xi^{*}{S_{h}}^{*}-\left(k\varpi +\alpha \left(1-\varpi \right)+\mu \right){E_{h}}^{*}=0\mathrm{\backslash vspace}1mm\\ \frac{d{I_{h}}^{*}}{dt}=\alpha \left(1-\varpi \right){E_{h}}^{*}+m\left(1-\varepsilon \right){T_{h}}^{*}-\left(\rho +l+\mu +\delta \right){I_{h}}^{*}=0 \end{array}$$

$$\begin{array}{c} \frac{d{T_{h}}^{*}}{dt}=k\varpi {E_{h}}^{*}+\rho {I_{h}}^{*}+\nu {A_{h}}^{*}-\left(\varpi \psi \varepsilon +m\left(1-\varepsilon \right)+\mu +\delta \right){T_{h}}^{*}=0\mathrm{\backslash vspace}1mm\\ \frac{d{U_{h}}^{*}}{dt}=\varpi \psi \varepsilon {T^{*}}_{h}-\mu {U_{h}}^{*}=0\mathrm{\backslash vspace}1mm\\ \frac{d{A_{h}}^{*}}{dt}=l{I_{h}}^{*}-\left(\nu +\mu +\delta \right){A_{h}}^{*}=0 \end{array}$$ (8)

The endemic equilibrium point is given as;


$${S_{h}}^{*}=\frac{\Lambda }{\left(\xi^{*}+\mu \right)},\text{\textbackslash{}hspace\{0.17em}\;\}{E_{h}}^{*}=\frac{\xi^{*}{S_{h}}^{*}}{W},\text{\textbackslash{}hspace\{0.17em}\;\}{I_{h}}^{*}=\frac{\alpha \left(1-\varpi \right){E_{h}}^{*}+m\left(1-\varepsilon \right){T_{h}}^{*}}{X}$$

$${T_{h}}^{*}=\frac{k\varpi {E_{h}}^{*}+\rho {I_{h}}^{*}+\nu {A_{h}}^{*}}{Y},\text{\textbackslash{}hspace\{0.17em}\;\;\}{U_{h}}^{*}=\frac{\varpi \psi \varepsilon {T_{h}}^{*}}{\mu },\text{\textbackslash{}hspace\{0.17em}\;\;\}{A_{h}}^{*}=\frac{l{I_{h}}^{*}}{Z}$$ (9)

Further substitutions and working gives;


$${T_{h}}^{*}=\frac{k\varpi \frac{\xi^{*}{S_{h}}^{*}}{W}+\rho {I_{h}}^{*}+\nu {A_{h}}^{*}}{Y}$$

$$\begin{array}{c} \Rightarrow {T_{h}}^{*}=\frac{XZk\varpi \xi^{*}{S_{h}}^{*}+\left(\rho Z\alpha \left(1-\varpi \right)\xi^{*}{S_{h}}^{*}+\rho ZWm\left(1-\varepsilon \right){T_{h}}^{*}\right)+\left(\nu l\alpha \left(1-\varpi \right)\xi^{*}{S_{h}}^{*}+\nu lWm\left(1-\varepsilon \right){T_{h}}^{*}\right)}{WXYZ}\mathrm{\backslash vspace}1mm\\ {T_{h}}^{*}\left(\left(WXZY\right)-Wm\left(1-\varepsilon \right)\left(\rho Z+\nu l\right)\right)=\xi^{*}{S_{h}}^{*}\left(XZk\varpi +\rho Z\alpha \left(1-\varpi \right)+\nu l\alpha \left(1-\varpi \right)\right)\\ {T_{h}}^{*}=\frac{\xi^{*}\Lambda \left(XZk\varpi +\rho Z\alpha \left(1-\varpi \right)+\nu l\alpha \left(1-\varpi \right)\right)}{W\left(\xi^{*}+\mu \right)\left(XYZ-m\left(1-\varepsilon \right)\left(\rho Z+\nu l\right)\right)} \end{array}$$

$${I_{h}}^{*}=\frac{\xi^{*}\Lambda \left(\alpha \left(1-\varpi \right)\left(\left(XZY\right)-m\left(1-\varepsilon \right)\left(\rho Z+\nu l\right)\right)+m\left(1-\varepsilon \right)\left(XZk\varpi +\rho Z\alpha \left(1-\varpi \right)+\nu l\alpha \left(1-\varpi \right)\right)\right)}{WX\left(\xi^{*}+\mu \right)\left(XYZ-m\left(1-\varepsilon \right)\left(\rho Z+\nu l\right)\right)}$$

$${A_{h}}^{*}=\frac{l\xi^{*}\Lambda \left(\alpha \left(1-\varpi \right)\left(\left(XZY\right)-m\left(1-\varepsilon \right)\left(\rho Z+\nu l\right)\right)+m\left(1-\varepsilon \right)\left(XZk\varpi +\rho Z\alpha \left(1-\varpi \right)+\nu l\alpha \left(1-\varpi \right)\right)\right)}{WXZ\left(\xi^{*}+\mu \right)\left(XYZ-m\left(1-\varepsilon \right)\left(\rho Z+\nu l\right)\right)}$$

Substituting in the force of infection gives;


$$\begin{array}{c} \xi^{*}=\frac{\beta \left(\left(1-\varepsilon \right){T_{h}}^{*}+\eta_{\text{1}}{I_{h}}^{*}+\eta_{\text{2}}{A_{h}}^{*}\right)}{N_{h}}\\ =\frac{\beta \left(\begin{array}{c} @l@\left(1-\varepsilon \right)\frac{\xi^{*}\Lambda \left(XZk\varpi +\rho Z\alpha \left(1-\varpi \right)+\nu l\alpha \left(1-\varpi \right)\right)}{W\left(\xi^{*}+\mu \right)\left(XYZ-m\left(1-\varepsilon \right)\left(\rho Z+\nu l\right)\right)}\\ +\eta_{\text{1}}\frac{\xi^{*}\Lambda \left(\alpha \left(1-\varpi \right)\left(\left(XZY\right)-m\left(1-\varepsilon \right)\left(\rho Z+\nu l\right)\right)+m\left(1-\varepsilon \right)\left(XZk\varpi +\rho Z\alpha \left(1-\varpi \right)+\nu l\alpha \left(1-\varpi \right)\right)\right)}{WX\left(\xi^{*}+\mu \right)\left(XYZ-m\left(1-\varepsilon \right)\left(\rho Z+\nu l\right)\right)}\\ +\eta_{\text{2}}\frac{l\xi^{*}\Lambda \left(\alpha \left(1-\varpi \right)\left(\left(XZY\right)-m\left(1-\varepsilon \right)\left(\rho Z+\nu l\right)\right)+m\left(1-\varepsilon \right)\left(XZk\varpi +\rho Z\alpha \left(1-\varpi \right)+\nu l\alpha \left(1-\varpi \right)\right)\right)}{WXZ\left(\xi^{*}+\mu \right)\left(XYZ-m\left(1-\varepsilon \right)\left(\rho Z+\nu l\right)\right)} \end{array}\right)}{N_{h}} \end{array}$$

$$\xi^{*}=\frac{\beta \left(\begin{array}{c} @l@\left(1-\varepsilon \right)\frac{\xi^{*}\Lambda \left(XZk\varpi +\rho Z\alpha \left(1-\varpi \right)+\nu l\alpha \left(1-\varpi \right)\right)}{W\left(\xi^{*}+\mu \right)\left(XYZ-m\left(1-\varepsilon \right)\left(\rho Z+\nu l\right)\right)}\\ +\eta_{\text{1}}\frac{\xi^{*}\Lambda \left(\alpha \left(1-\varpi \right)\left(\left(XZY\right)-m\left(1-\varepsilon \right)\left(\rho Z+\nu l\right)\right)+m\left(1-\varepsilon \right)\left(XZk\varpi +\rho Z\alpha \left(1-\varpi \right)+\nu l\alpha \left(1-\varpi \right)\right)\right)}{WX\left(\xi^{*}+\mu \right)\left(XYZ-m\left(1-\varepsilon \right)\left(\rho Z+\nu l\right)\right)}\\ +\eta_{\text{2}}\frac{l\xi^{*}\Lambda \left(\alpha \left(1-\varpi \right)\left(\left(XZY\right)-m\left(1-\varepsilon \right)\left(\rho Z+\nu l\right)\right)+m\left(1-\varepsilon \right)\left(XZk\varpi +\rho Z\alpha \left(1-\varpi \right)+\nu l\alpha \left(1-\varpi \right)\right)\right)}{WXZ\left(\xi^{*}+\mu \right)\left(XYZ-m\left(1-\varepsilon \right)\left(\rho Z+\nu l\right)\right)} \end{array}\right)}{N_{h}}$$

$$\mathrm{\backslash footnotesize}\begin{array}{c} \left(\xi^{*}\right)=\frac{\beta \Lambda \left(\begin{array}{c} @l@\left(1-\varepsilon \right)XZ\left(XZk\varpi +\rho Z\alpha \left(1-\varpi \right)+\nu l\alpha \left(1-\varpi \right)\right)\\ +\eta_{\text{1}}\left(\alpha \;\left(1-\varpi \right)Z\;\left(\left(XZY\right)\;-\;m\left(1-\varepsilon \right)\left(\rho Z+\nu l\right)\right)+m\left(1-\varepsilon \;\right)\;\left(XZk\varpi \;+\;\rho Z\alpha \left(1-\varpi \right)\;+\;\nu l\alpha \;\left(1-\varpi \right)\right)\right)\\ \;+\;\eta_{\text{2}}l\left(\alpha \left(1-\varpi \right)\left(\left(XZY\right)-m\left(1-\varepsilon \right)\left(\rho Z\;+\;\nu l\right)\right)\;+\;m\left(1-\varepsilon \right)\left(XZk\varpi \;+\;\rho Z\alpha \left(1-\varpi \right)\;+\;\nu l\alpha \left(1-\varpi \right)\right)\right) \end{array}\right)}{N_{h}WXZ\left(XYZ-m\left(1-\varepsilon \right)\left(\rho Z+\nu l\right)\right)}-\frac{\mu WXZ\left(XYZ-m\left(1-\varepsilon \right)\left(\rho Z+\nu l\right)\right)}{WXZ\left(XYZ-m\left(1-\varepsilon \right)\left(\rho Z+\nu l\right)\right)}\\ \xi^{*}=\frac{\beta \Lambda \left(\begin{array}{c} @l@\left(X^{\text{2}}Z^{\text{2}}\left(1-\varepsilon \right)k\varpi +XZ\left(1-\varepsilon \right)\alpha \left(1-\varpi \right)\left(\rho Z+\nu l\right)\right)\\ +\eta_{\text{1}}Z\left(\alpha \left(1-\varpi \right)\left(\left(XZY\right)+XZm\left(1-\varepsilon \right)k\varpi \right)\right)\\ +\eta_{\text{2}}l\left(\alpha \left(1-\varpi \right)\left(XZY\right)+XZm\left(1-\varepsilon \right)k\varpi \right) \end{array}\right)}{N_{h}WXZ\left(XYZ-m\left(1-\varepsilon \right)\left(\rho Z+\nu l\right)\right)}-\mu \\ \xi^{*}=\mu \left(\frac{\beta \Lambda \left(\left(XZ\left(1-\varepsilon \right)k\varpi +\left(1-\varepsilon \right)\alpha \left(1-\varpi \right)\left(\rho Z+\nu l\right)\right)+\eta_{\text{1}}Z\left(\alpha \left(1-\varpi \right)\left(\left(Y\right)+m\left(1-\varepsilon \right)k\varpi \right)\right)+\eta_{\text{2}}l\left(\alpha \left(1-\varpi \right)\left(Y\right)+m\left(1-\varepsilon \right)k\varpi \right)\right)}{\mu N_{h}W\left(XYZ-m\left(1-\varepsilon \right)\left(\rho Z+\nu l\right)\right)}-1\right) \end{array}$$

$$\xi^{*}=\mu \left(R_{\varepsilon }-1\right)$$ (10)

Thus, the force of infection at steady-state $\xi^{*}$ is positive only if $R_{\varepsilon }>1$.

Therefore, the model system (1) has a unique endemic equilibrium whenever $R_{\varepsilon }>1$

## 4. Optimal control model

In our optimal control of HIV infection, we extend our model (1) by incorporating three distinct time-dependent control measures. Optimal control analysis helps identify the best control measure to adopt in the eradication or control of the disease in the community at a specified period [15,21,22]. The three single control strategies considered are;

1. Pre-contact preventive measures $u_{\text{1}}$, such as those that protect the susceptible from contracting HIV infection.
2. Post-contact preventive measure $u_{\text{2}},$aimed at protecting from contracting HIV infection after an effective exposure to the disease. This is the use of Post-Exposure Prophylaxis (PEP) intervention. This is usually commenced within 72 hours after an effective exposure to the virus to prevent infection [16].
3. Treatment control measures $u_{\text{3}}$, are used on pre-AIDS and AIDS individuals to reduce the viral load and stop or slow the progression.

Incorporating these control measures in our model (1) gives:


$$\left\{ \begin{array}{c} @l@\frac{dS_{h}}{dt}=\Lambda -\left(\left(1-u_{\text{1}}\left(t\right)\right)\frac{\beta \left(\left(1-\varepsilon \right)T_{h}+\eta_{\text{1}}I_{h}+\eta_{\text{2}}A_{h}\right)}{N_{h}}+\mu \right)S_{h}\\ \frac{dE_{h}}{dt}=\left(1-u_{\text{1}}\left(t\right)\right)\frac{\beta \left(\left(1-\varepsilon \right)T_{h}+\eta_{\text{1}}I_{h}+\eta_{\text{2}}A_{h}\right)}{N_{h}}S_{h}-\left(ku_{\text{2}}\left(t\right)+\alpha \left(1-u_{\text{2}}\left(t\right)\right)+\mu \right)E_{h}\\ \frac{dI_{h}}{dt}=\alpha \left(1-u_{\text{2}}\left(t\right)\right)E_{h}+m\left(1-\varepsilon \right)T_{h}-\left(u_{\text{3}}\left(t\right)\rho +\left(1-u_{\text{3}}\left(t\right)\right)l+\mu +\delta \right)I_{h}\\ \frac{dT_{h}}{dt}=ku_{\text{2}}\left(t\right)E_{h}+u_{\text{3}}\left(t\right)\rho I_{h}+u_{\text{3}}\left(t\right)\nu A_{h}-\left(u_{\text{2}}\left(t\right)\psi \varepsilon +m\left(1-\varepsilon \right)+\mu +\delta \right)T_{h}\\ \frac{dU_{h}}{dt}=u_{\text{2}}\left(t\right)\psi \varepsilon T_{h}-\left(\mu \right)U_{h}\\ \frac{dA_{h}}{dt}=\left(1-u_{\text{3}}\left(t\right)\right)lI_{h}-\left(u_{\text{3}}\left(t\right)\nu +\mu +\delta \right)A_{h} \end{array}\right.$$ (11)

where $\varpi =u_{\text{2}}$and with the initial conditions, $S_{h}>0,E_{h}>0,I_{h}>0,T_{h}>0,U_{h}>0,A_{h}>0$ AIDS patients are treated with ART to have their HIV viral loads lowered and progress to the Treatment class at the rate v

Our goal is to minimize the population of the under-3-day Exposed $\left(E_{h}\right)$, the pre-AIDS population $\left(I_{h}\right)$, the population on treatment $\left(T_{h}\right)$, and the AIDS $\left(A_{h}\right)$ population. We also, seek to increase the number of those who were Uninfected $\left(U_{h}\right)$, after an exposure to the virus, and consider the cost involved in using these control strategies.

From Pontryagin [23] and Fleming [24], we have the objective function defined as:


$$J\left(u_{\text{1}},u_{\text{2}},u_{\text{3}}\right)=\int_{\text{0}}^{tf}\left(B_{\text{1}}E_{h}+B_{\text{2}}I_{h}+B_{\text{3}}T_{h}+B_{\text{4}}A_{h}+C_{\text{1}}\frac{{u_{\text{1}}}^{\text{2}}}{\text{2}}+C_{\text{2}}\frac{{u_{\text{2}}}^{\text{2}}}{\text{2}}+C_{\text{3}}\frac{{u_{\text{3}}}^{\text{2}}}{\text{2}}\right)dt,$$ (12)

where $B_{\text{1}},B_{\text{2}},B_{\text{3}},B_{\text{4}}$ are all positive weight constants of the under 3 days-Exposed, pre-AIDS, Treatment and AIDS classes, respectively. Also, $C_{\text{1}}\frac{{u_{\text{1}}}^{\text{2}}}{\text{2}},C_{\text{2}}\frac{{u_{\text{2}}}^{\text{2}}}{\text{2}}$and $C_{\text{3}}\frac{{u_{\text{3}}}^{\text{2}}}{\text{2}}$ are the cost control functions. The constants $C_{\text{1}},C_{\text{2}},C_{\text{3}}$ are measures of the relative cost of the interventions associated with the controls $u_{i}$ [25]. The cost function is quadratic because it is nonlinear [10]. We aim to find the optimal control of ${u_{\text{1}}}^{*}\left(t\right),{u_{\text{2}}}^{*}\left(t\right)$ and ${u_{\text{3}}}^{*}\left(t\right)$ such that $J\left({u_{\text{1}}}^{*}\left(t\right),{u_{\text{2}}}^{*}\left(t\right),{u_{\text{3}}}^{*}\left(t\right)\right)=min\left\{\left(u_{\text{1}},u_{\text{2}},u_{\text{3}}\right):u_{\text{1}},u_{\text{2}},u_{\text{3}}\in \Delta \right\}$, where Δ is a non-empty control, subject to the optimal control model set defined as $\Delta =\{u_{i}:0\leq u_{i}\left(t\right)\leq 1$, Lebesgue measurable $t=\left[0,t_{f}\right]$ for i=1,2,3}, representing the control set with respect to the initial conditions.

To show that the optimal control problem exists, we employ Pontryagin’s maximum principle [23], which provides the necessary condition for the existence optimal controls$u^{*}=\left(u_{\text{1}},u_{\text{2}},u_{\text{3}}\right)$. By this principle, we have the Hamiltonian function (H) defined as;


$$\begin{array}{c} H\left(S_{h},E_{h},I_{h},T_{h},U_{h},A_{h}\right)=B_{\text{1}}E_{h}+B_{\text{2}}I_{h}+B_{\text{3}}T_{h}+B_{\text{4}}A_{h}+\frac{\text{1}}{\text{2}}\left(C_{\text{1}}{u_{\text{1}}}^{\text{2}}+C_{\text{2}}{u_{\text{2}}}^{\text{2}}+C_{\text{3}}{u_{\text{3}}}^{\text{2}}\right)\\ \text{\textbackslash{}hspace\{1em}\;\;\;\;\;\;\;\;\;\}+\lambda_{\text{1}}\frac{dS_{h}}{dt}+\lambda_{\text{2}}\frac{dE_{h}}{dt}+\lambda_{\text{3}}\frac{dI_{h}}{dt}+\lambda_{\text{4}}\frac{dT_{h}}{dt}+\lambda_{\text{5}}\frac{dU_{h}}{dt}+\lambda_{\text{6}}\frac{dA_{h}}{dt} \end{array}$$ (13)

Where $\lambda_{i},i=1,2,3,4,5,6$ are the adjoint variables associated with the state variables of the optimal control model (11)

From Peter O. *et al.* [20], we establish the following results.

**Theorem 3** Let ${S_{h}}^{*},{E_{h}}^{*},{I_{h}}^{*},{T_{h}}^{*},{U_{h}}^{*},{A_{h}}^{*}$, be the optimal state solutions associated with the optimal control ${u_{\text{1}}}^{*},{u_{\text{2}}}^{*},{u_{\text{3}}}^{*}$, for the optimal problem in (11). There exist adjoint functions $\lambda_{i}$, which satisfy the system (13) with the transversal conditions $\lambda_{i}\left(t_{f}\right)=0$, for i=1,2,3,3,4,5,6, and the control variables $\left({u_{\text{1}}}^{*},{u_{\text{2}}}^{*},{u_{\text{3}}}^{*}\right)$.

Proof:

From the second condition of Pontryagin’s maximum principle (PMP), there exist adjoint variables, $\lambda_{i}$, i=1,2,3,3,4,5,6 which satisfy the following canonical equations: $\frac{d\lambda_{\text{1}}}{dt}=-\frac{dH}{dS_{h}},$$\frac{d\lambda_{\text{2}}}{dt}=-\frac{dH}{dE_{h}},$$\frac{d\lambda_{\text{3}}}{dt}=-\frac{dH}{dI_{h}},$$\frac{d\lambda_{\text{4}}}{dt}=-\frac{dH}{dT_{h}},\frac{d\lambda_{\text{5}}}{dt}=-\frac{dH}{dU_{h}},$ and $\frac{d\lambda_{\text{6}}}{dt}=-\frac{dH}{dA_{h}}$

Differentiating the Hamiltonian function (13) with respect to the state variables $S_{h},E_{h},I_{h},T_{h},U_{h}$ and $A_{h}$, we have:


$$\begin{array}{c} \frac{d\lambda_{\text{1}}}{dt}=-\frac{\partial H}{\partial S}=-\left(\left(\begin{array}{c} @l@-\lambda_{\text{1}}\left(1-u_{\text{1}}\left(t\right)\right)\left(\frac{\beta \left(\left(1-\varepsilon \right)T_{h}+\eta_{\text{1}}I_{h}+\eta_{\text{2}}A_{h}\right)}{N_{h}}-\frac{\beta \left(\left(1-\varepsilon \right)T_{h}+\eta_{\text{1}}I_{h}+\eta_{\text{2}}A_{h}\right)S}{{N_{h}}^{\text{2}}}\right)-\lambda_{\text{1}}\mu \\ +\lambda_{\text{2}}\left(\left(1-u_{\text{1}}\left(t\right)\right)\frac{\beta \left(\left(1-\varepsilon \right)T_{h}+\eta_{\text{1}}I_{h}+\eta_{\text{2}}A_{h}\right)}{N_{h}}-\frac{\beta \left(\left(1-\varepsilon \right)T_{h}+\eta_{\text{1}}I_{h}+\eta_{\text{2}}A_{h}\right)S}{{N_{h}}^{\text{2}}}\right) \end{array}\right)\right)\\ \text{\textbackslash{}hspace\{1em}\;\;\;\}=\left(\lambda_{\text{1}}-\lambda_{\text{2}}\right)\left(1-u_{\text{1}}\left(t\right)\right)\left(\frac{\beta \left(\left(1-\varepsilon \right)T_{h}+\eta_{\text{1}}I_{h}+\eta_{\text{2}}A_{h}\right)}{N_{h}}-\frac{\beta \left(\left(1-\varepsilon \right)T_{h}+\eta_{\text{1}}I_{h}+\eta_{\text{2}}A_{h}\right)S}{{N_{h}}^{\text{2}}}\right)+\lambda_{\text{1}}\mu \\ \text{\textbackslash{}hspace\{1em}\;\;\;\}=\left(\lambda_{\text{1}}-\lambda_{\text{2}}\right)\left(1-u_{\text{1}}\left(t\right)\right)\left(\frac{\beta \left(\left(1-\varepsilon \right)T_{h}+\eta_{\text{1}}I_{h}+\eta_{\text{2}}A_{h}\right)}{N_{h}}\right)\left(1-\frac{S}{N_{h}}\right)+\lambda_{\text{1}}\mu \end{array}$$

$$\begin{array}{c} \frac{d\lambda_{\text{2}}}{dt}=-\frac{\partial H}{\partial E}=-\left(\begin{array}{c} @l@\left(B_{\text{1}}\right)+\lambda_{\text{1}}\left(1-u_{\text{1}}\left(t\right)\right)\frac{\beta \left(\left(1-\varepsilon \right)T_{h}+\eta_{\text{1}}I_{h}+\eta_{\text{2}}A_{h}\right)S}{{N_{h}}^{\text{2}}}-\lambda_{\text{2}}\left(1-u_{\text{1}}\left(t\right)\right)\frac{\beta \left(\left(1-\varepsilon \right)T_{h}+\eta_{\text{1}}I_{h}+\eta_{\text{2}}A_{h}\right)S}{{N_{h}}^{\text{2}}}\\ -\lambda_{\text{2}}\left(ku_{\text{2}}\left(t\right)+\alpha \left(1-u_{\text{2}}\left(t\right)\right)+\mu \right)+\lambda_{\text{3}}\alpha \left(1-u_{\text{2}}\left(t\right)\right)+\lambda_{\text{4}}ku_{\text{2}}\left(t\right) \end{array}\right)\\ \text{\textbackslash{}hspace\{1em}\;\;\}=-B_{\text{1}}+\left(\lambda_{\text{2}}-\lambda_{\text{1}}\right)\left(1-u_{\text{1}}\left(t\right)\right)\frac{\beta \left(\left(1-\varepsilon \right)T_{h}+\eta_{\text{1}}I_{h}+\eta_{\text{2}}A_{h}\right)S}{{N_{h}}^{\text{2}}}+\left(\lambda_{\text{2}}-\lambda_{\text{4}}\right)kuu_{\text{2}}\left(t\right)+\left(\lambda_{\text{2}}-\lambda_{\text{3}}\right)\alpha \left(1-u_{\text{2}}\left(t\right)\right)+\lambda_{\text{2}}\mu \end{array}$$

$$\begin{array}{c} \frac{d\lambda_{\text{3}}}{dt}=-\frac{\partial H}{\partial I}=-\left(\begin{array}{c} @l@\left(B_{\text{2}}\right)-\lambda_{\text{1}}\left(1-u_{\text{1}}\left(t\right)\right)\left(\frac{\beta \left(\eta_{\text{1}}\right)S}{N_{h}}-\frac{\beta \left(\left(1-\varepsilon \right)T_{h}+\eta_{\text{1}}I_{h}+\eta_{\text{2}}A_{h}\right)S}{{N_{h}}^{\text{2}}}\right)\\ +\lambda_{\text{2}}\left(1-u_{\text{1}}\left(t\right)\right)\left(\frac{\beta \left(\eta_{\text{1}}\right)S}{N_{h}}-\frac{\beta \left(\left(1-\varepsilon \right)T_{h}+\eta_{\text{1}}I_{h}+\eta_{\text{2}}A_{h}\right)S}{{N_{h}}^{\text{2}}}\right)-\lambda_{\text{3}}\left(u_{\text{3}}\left(t\right)\rho +\left(1-u_{\text{3}}\left(t\right)\right)l+\mu +\delta \right)\\ +\lambda_{\text{4}}u_{\text{3}}\left(t\right)\rho +\lambda_{\text{6}}\left(1-u_{\text{3}}\left(t\right)\right)l \end{array}\right)\\ \text{\textbackslash{}hspace\{1em}\;\}=-B_{\text{2}}+\left(\lambda_{\text{1}}-\lambda_{\text{2}}\right)\left(1-u_{\text{1}}\left(t\right)\right)\frac{\beta S}{N_{h}}\left(\eta_{\text{1}}-\frac{\left(\left(1-\varepsilon \right)T_{h}+\eta_{\text{1}}I_{h}+\eta_{\text{2}}A_{h}\right)}{N_{h}}\right)\\ \text{\textbackslash{}hspace\{1em}\;\;\}+\left(\lambda_{\text{3}}-\lambda_{\text{4}}\right)u_{\text{3}}\left(t\right)\rho +\left(\lambda_{\text{3}}-\lambda_{\text{6}}\right)\left(1-u_{\text{3}}\left(t\right)\right)l+\lambda_{\text{3}}\left(\mu +\delta \right) \end{array}$$

$$\begin{array}{c} \frac{d\lambda_{\text{4}}}{dt}=-\frac{\partial H}{\partial T_{h}}=-\left(\begin{array}{c} @l@B_{\text{3}}-\lambda_{\text{1}}\left(1-u_{\text{1}}\left(t\right)\right)\left(\frac{\beta \left(1-\varepsilon \right)S}{N_{h}}-\frac{\beta \left(\left(1-\varepsilon \right)T_{h}+\eta_{\text{1}}I_{h}+\eta_{\text{2}}A_{h}\right)S}{{N_{h}}^{\text{2}}}\right)\\ +\lambda_{\text{2}}\left(1-u_{\text{1}}\left(t\right)\right)\left(\frac{\beta \left(1-\varepsilon \right)S}{N_{h}}-\frac{\beta \left(\left(1-\varepsilon \right)T_{h}+\eta_{\text{1}}I_{h}+\eta_{\text{2}}A_{h}\right)S}{{N_{h}}^{\text{2}}}\right)\\ +\lambda_{\text{3}}m\left(1-\varepsilon \right)-\lambda_{\text{4}}\left(u_{\text{2}}\left(t\right)\psi \varepsilon +m\left(1-\varepsilon \right)+\mu +\delta \right)+\lambda_{\text{5}}u_{\text{2}}\left(t\right)\psi \varepsilon \end{array}\right)\\ \text{\textbackslash{}hspace\{1em}\;\}=-B_{\text{3}}+\left(\lambda_{\text{1}}-\lambda_{\text{2}}\right)\left(1-u_{\text{1}}\left(t\right)\right)\frac{\beta S}{N_{h}}\left(\left(1-\varepsilon \right)-\frac{\left(\left(1-\varepsilon \right)T_{h}+\eta_{\text{1}}I_{h}+\eta_{\text{2}}A_{h}\right)}{N_{h}}\right)\\ \text{\textbackslash{}hspace\{1em}\;\;\}+\left(\lambda_{\text{4}}-\lambda_{\text{3}}\right)m\left(1-\varepsilon \right)+\left(\lambda_{\text{4}}-\lambda_{\text{5}}\right)u_{\text{2}}\left(t\right)\psi \varepsilon +\lambda_{\text{4}}\left(\mu +\delta \right) \end{array}$$

$$\begin{array}{c} \frac{d\lambda_{\text{5}}}{dt}=-\frac{\partial H}{\partial U_{h}}=-\left(\begin{array}{c} @l@-\lambda_{\text{1}}\left(1-u_{\text{1}}\left(t\right)\right)\frac{\beta \left(\left(1-\varepsilon \right)T_{h}+\eta_{\text{1}}I_{h}+\eta_{\text{2}}A_{h}\right)S}{{N_{h}}^{\text{2}}}+\\ \lambda_{\text{2}}\left(1-u_{\text{1}}\left(t\right)\right)\frac{\beta \left(\left(1-\varepsilon \right)T_{h}+\eta_{\text{1}}I_{h}+\eta_{\text{2}}A_{h}\right)S}{{N_{h}}^{\text{2}}}-\lambda_{\text{5}}\mu \end{array}\right)\\ \text{\textbackslash{}hspace\{1em}\;\;\;\}=\left(\lambda_{\text{1}}-\lambda_{\text{2}}\right)\left(1-u_{\text{1}}\left(t\right)\right)\frac{\beta \left(\left(1-\varepsilon \right)T_{h}+\eta_{\text{1}}I_{h}+\eta_{\text{2}}A_{h}\right)S}{{N_{h}}^{\text{2}}}+\lambda_{\text{5}}\mu \end{array}$$

$$\begin{array}{c} \frac{d\lambda_{\text{6}}}{dt}=-\frac{\partial H}{\partial A_{h}}=-\left(\begin{array}{c} @l@B_{\text{4}}-\lambda_{\text{1}}\left(1-u_{\text{1}}\left(t\right)\right)\left(\frac{\beta \eta_{\text{2}}S}{N_{h}}-\frac{\beta \left(\left(1-\varepsilon \right)T_{h}+\eta_{\text{1}}I_{h}+\eta_{\text{2}}A_{h}\right)S}{{N_{h}}^{\text{2}}}\right)\\ +\lambda_{\text{2}}\left(1-u_{\text{1}}\left(t\right)\right)\left(\frac{\beta \eta_{\text{2}}S}{N_{h}}-\frac{\beta \left(\left(1-\varepsilon \right)T_{h}+\eta_{\text{1}}I_{h}+\eta_{\text{2}}A_{h}\right)S}{{N_{h}}^{\text{2}}}\right)+\lambda_{\text{4}}u_{\text{3}}\left(t\right)\nu -\lambda_{\text{6}}\left(u_{\text{3}}\left(t\right)\nu +\mu +\delta \right) \end{array}\right)\\ \text{\textbackslash{}hspace\{1em}\;\}=-B_{\text{4}}+\left(\lambda_{\text{1}}-\lambda_{\text{2}}\right)\left(1-u_{\text{1}}\left(t\right)\right)\frac{\beta S}{N_{h}}\left(\eta_{\text{2}}-\frac{\left(\left(1-\varepsilon \right)T_{h}+\eta_{\text{1}}I_{h}+\eta_{\text{2}}A_{h}\right)}{N_{h}}\right)+\left(\lambda_{\text{6}}-\lambda_{\text{4}}\right)u_{\text{3}}\left(t\right)\nu +\lambda_{\text{6}}\left(\mu +\delta \right) \end{array}$$

with transversal conditions$\lambda_{i}\left(t_{f}\right)=0,$ i=1,2,3,3,4,5,6

We establish the optimal control of the control variable set, where $u_{i}=\left(0,1\right),\text{\textbackslash{}hspace\{1em}\}i=1,2,3$ by differentiating the Hamiltonian H in (13) at the steady state, with respect to the control variables $\left(u_{\text{1}},u_{\text{2}},u_{\text{3}}\right)$. That is $\frac{dH}{du_{\text{1}}}{|}_{u_{\text{1}}={u_{\text{1}}}^{*}}=0$, $\frac{dH}{du_{\text{2}}}{|}_{u_{\text{2}}={u_{\text{2}}}^{*}}=0$, $\frac{dH}{du_{\text{3}}}{|}_{u_{\text{3}}={u_{\text{3}}}^{*}}=0$


$$\frac{dH}{du_{\text{1}}}=C_{\text{1}}u_{\text{1}}+\lambda_{\text{1}}\frac{\beta \left(\left(1-\varepsilon \right)T_{h}+\eta_{\text{1}}I_{h}+\eta_{\text{2}}A_{h}\right)S}{N_{h}}-\lambda_{\text{2}}\frac{\beta \left(\left(1-\varepsilon \right)T_{h}+\eta_{\text{1}}I_{h}+\eta_{\text{2}}A_{h}\right)S}{N_{h}}=0$$

$${u_{\text{1}}}^{*}=\left(\lambda_{\text{2}}-\lambda_{\text{1}}\right)\frac{\beta \left(\left(1-\varepsilon \right)T_{h}+\eta_{\text{1}}I_{h}+\eta_{\text{2}}A_{h}\right)S}{C_{\text{1}}N_{h}}$$

$$\begin{array}{c} \frac{dH}{du_{\text{2}}}=C_{\text{2}}u_{\text{2}}-\lambda_{\text{2}}\left(k-\alpha \right)E_{h}-\lambda_{\text{3}}\alpha E_{h}+\lambda_{\text{4}}\left(kE_{h}-\psi \varepsilon T_{h}\right)+\lambda_{\text{5}}\psi \varepsilon T_{h}=0\\ \Rightarrow {u_{\text{2}}}^{*}=\frac{\left(\lambda_{\text{3}}-\lambda_{\text{2}}\right)\alpha E_{h}+\left(\lambda_{\text{2}}-\lambda_{\text{4}}\right)kE_{h}+\left(\lambda_{\text{4}}-\lambda_{\text{5}}\right)\psi \varepsilon T_{h}}{C_{\text{2}}} \end{array}$$

$$\begin{array}{c} \frac{dH}{du_{\text{3}}}=C_{\text{3}}u_{\text{3}}-\lambda_{\text{3}}\left(\rho -l\right)I_{h}+\lambda_{\text{4}}\left(\rho I_{h}+\nu A_{h}\right)-\lambda_{\text{6}}\left(lI_{h}+vA_{h}\right)=0\\ \Rightarrow {u_{\text{3}}}^{*}=\frac{\left(\lambda_{\text{3}}-\lambda_{\text{4}}\right)\rho I_{h}+\left(\lambda_{\text{6}}-\lambda_{\text{3}}\right)lI_{h}+\left(\lambda_{\text{6}}-\lambda_{\text{4}}\right)vA_{h}}{C_{\text{3}}} \end{array}$$

Therefore:


$${u_{\text{1}}}^{*}=max\left\{0,\left(\left(\lambda_{\text{2}}-\lambda_{\text{1}}\right)\frac{\beta \left(\left(1-\varepsilon \right)T_{h}+\eta_{\text{1}}I_{h}+\eta_{\text{2}}A_{h}\right)S}{C_{\text{1}}N_{h}}\right)\right\}$$

$${u_{\text{2}}}^{*}=max\left\{0,\left(\frac{\left(\lambda_{\text{3}}-\lambda_{\text{2}}\right)\alpha E_{h}+\left(\lambda_{\text{2}}-\lambda_{\text{4}}\right)kE_{h}+\left(\lambda_{\text{4}}-\lambda_{\text{5}}\right)\psi \varepsilon T_{h}}{C_{\text{2}}}\right)\right\}$$

$${u_{\text{3}}}^{*}=max\left\{0,\left(\frac{\left(\lambda_{\text{3}}-\lambda_{\text{4}}\right)\rho I_{h}+\left(\lambda_{\text{6}}-\lambda_{\text{3}}\right)lI_{h}+\left(\lambda_{\text{6}}-\lambda_{\text{4}}\right)vA_{h}}{C_{\text{3}}}\right)\right\}$$

## 5. Numerical simulation of the optimal control model and discussion

In this section, we investigate the impact of different optimal control measures and combined control measures to find the one that can quickly eradicate or curtail the spread of HIV/AIDS. The most effective cost strategy suitable for implementation among the various combinations of these control strategies is discussed. This is very necessary because disease eradication in a given population is always cost-intensive. When recommending an intervention strategy for disease eradication, cost-effectiveness information on the interventions is needed for the optimal allocation of available resources.

Numerical simulations have been useful in determining the impact of these control measures on an ideal control model. It is useful in describing the dynamical behavior of a given system over time for different parameter values and conditions. Thus, we use Numerical simulation to illustrate the effect of these control measures and their combined strategies, using fourth-order Runge-Kutta forward–backward sweep simulations.

To this effect, we investigate the effect of optimal control strategies on the spread of HIV with the following categorization:

Strategy 1:Pre-contact prevention strategy $\left(u_{\text{1}}\right)$
Strategy 2:Post-contact prevention strategy $\left(u_{\text{2}}\right)$
Strategy 3:Control effort using only Anti-Retroviral treatment on the infected $\left(u_{\text{3}}\right)$
Paired strategies (4, 5, and 6): These are control efforts by pairing the strategies 1, 2, and 3. They are; $\left(u_{\text{1}},u_{\text{2}}\right)$, $\left(u_{\text{1}},u_{\text{3}}\right)$ and $\left(u_{\text{2}},u_{\text{3}}\right)$ respectively.
Strategy 7: Applying the first three strategies simultaneously $\left(u_{\text{1}},u_{\text{2}},u_{\text{2}}\right)$

The simulation’s initial conditions were taken from Tigabu Kasia Ayele *et al* [21], which are the approximated data of the HIV/AIDS rampage for the year 2019 in Ethiopia. The initial size of the populations has a fixed final time, $t_{f}=10years$. $S_{h}\left(0\right)=1,000,000$, $E_{h}\left(0\right)=144,900$, $I_{h}\left(0\right)=363,400$, $T_{h}\left(0\right)=448,000$, $U_{h}\left(0\right)=0$, $A_{h}\left(0\right)=181,700$ representing a total population of N=2,138,000.

Table 2 contains the parameter values used in the simulation, taken from the references cited therein. We have assumed some of them just for numerical purposes.

**Table 2:** Model parameter values.

Parameters	Values	Source
Λ	2000	Assumed
β	0.866/year	[6]
ε	0.02	Assumed
$\eta_{\text{1}}$	0.8	[21]
$\eta_{\text{2}}$	1.05	[21]
μ	0.02/year	[26]
l	0.1/year	[27]
m	0.75/year	[28]
δ	1/year	[29]
ψ	0.8	[17]
α	0.333	[10]
v	0.1	[29]
ρ	0.5/year	[15]
k	0.03	Assumed

Unfortunately, we do not have good data on the coefficients of the costs associated with the control variables and infected persons. However, while we assume the positive wait constants in the objective function to be equal to ensure that none of the four terms is dominant during simulation, we cannot say the same for weight constants relating to the costs of control implementation. The cost associated with $u_{\text{1}}$, includes the cost of enlightenment through different channels like public campaigns, media, schools, and health centers. Cost of the provision of PrEP drugs and other protective devices like condoms. $u_{\text{2}}$ requires most of the cost associated with $u_{\text{1}}$ and cost of PEP, instead of PrEP drugs, in every health center and facility, which should be readily available and subsidized for the populace. $u_{\text{3}}$’s cost is associated with the cost of enlightenment, ART drugs, and hospitalization. Thus, we assume $B_{\text{1}}=B_{\text{2}}=B_{\text{3}}=B_{\text{4}}=1$ and $C_{\text{1}}\text{\textbackslash{}hspace\{0.33em}\}=20,\text{\textbackslash{}hspace\{0.33em}\}C_{\text{2}}\text{\textbackslash{}hspace\{0.33em}\}=15,\text{\textbackslash{}hspace\{0.33em}\}C_{\text{3}}\text{\textbackslash{}hspace\{0.33em}\}=18$. Note that the weight constants are chosen based on assumptions and, as such, can fluctuate depending on different countries’ realities.

Table 3 shows the possible classifications of these control strategies under the outlined control measures.

**Table 3:** Control measures.

Control Measures	Strategies
Single control:	Strategy 1:$u_{\text{1}}\neq 0,\text{\textbackslash{}hspace\{0.33em}\}u_{\text{2}}=0,\text{\textbackslash{}hspace\{0.33em}\}u_{\text{3}}=0$ Strategy 2:$u_{\text{1}}=0,\text{\textbackslash{}hspace\{0.33em}\}u_{\text{2}}\neq 0,\text{\textbackslash{}hspace\{0.33em}\}u_{\text{3}}=0$ Strategy 3:$u_{\text{1}}=0,\text{\textbackslash{}hspace\{0.33em}\}u_{\text{2}}=0,\text{\textbackslash{}hspace\{0.33em}\}u_{\text{3}}\neq 0$
Double Control	Strategy 4:$u_{\text{1}}\neq 0,\text{\textbackslash{}hspace\{0.33em}\}u_{\text{2}}\neq 0,\text{\textbackslash{}hspace\{0.33em}\}u_{\text{3}}=0$ Strategy 5:$u_{\text{1}}\neq 0,\text{\textbackslash{}hspace\{0.33em}\}u_{\text{2}}=0,\text{\textbackslash{}hspace\{0.33em}\}u_{\text{3}}\neq 0$ Strategy 6:$u_{\text{1}}=0,\text{\textbackslash{}hspace\{0.33em}\}u_{\text{2}}\neq 0,\text{\textbackslash{}hspace\{0.33em}\}u_{\text{3}}\neq 0$
Triple Control	Strategy 7:$u_{\text{1}}\neq 0,\text{\textbackslash{}hspace\{0.33em}\}u_{\text{2}}\neq 0,\text{\textbackslash{}hspace\{0.33em}\}u_{\text{3}}\neq 0$

As shown in Table 3, each control measure is further divided into various strategies. We therefore simulate these strategies under each of the control measures. In our simulations, the under- 3-day HIV-Exposed class is referred to as the Exposed Class.

**Fig 2:**
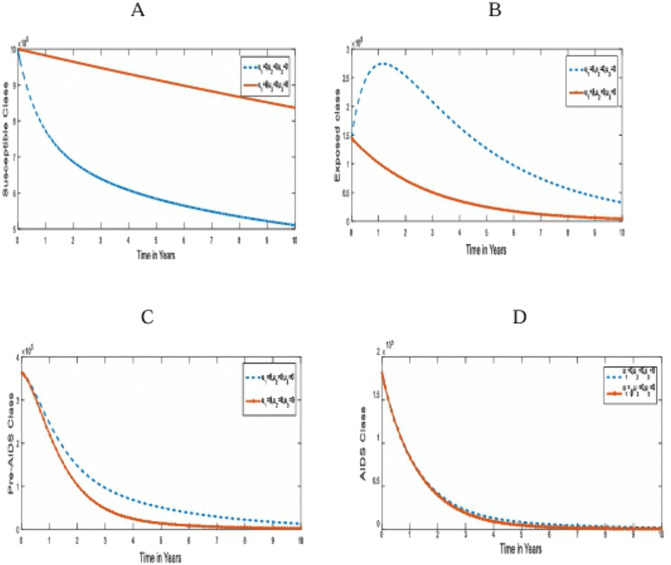
Simulations of the effect of Strategy 1 on the optimal control model. **A.** Susceptible population; **B.** under-3-day Exposed population; **C.** Pre-AIDS population; **D.** AIDS Population for $u_{\text{2}},u_{\text{3}}=0$.

**Strategy 1**: The optimal use of pre-contact preventive strategies $\left(u_{\text{1}}\right)$ only, for the control of transmission of HIV/AIDS in humans [30] . Fig 2 compares the population dynamics of the susceptible, under-3-day exposed, Pre-AIDS, and AIDS classes on the optimal application of strategy 1 and when it is absent.

In Fig 2A, there is a drastic increase in the population size of the susceptible class. This is because pre-contact prevention measures were aimed at protecting the population from initial contact with the virus, thereby reducing the population of the under-3-day Exposed and the Pre-AIDS population, as seen in Fig 2B and 2C. However, Fig 2D shows that the pre-contact prevention strategy alone has no appreciable effect on the individuals living with AIDS since they are already living with the virus, and there is no treatment. This intervention averted 2.4592 x 10^6^ infected individuals. With this single control strategy, we notice that the infected populations tend to zero from the 7th year of its optimal implementation, as shown in Fig 2C.

**Strategy 2**: The optimal use of post-contact preventive strategy $\left(u_{\text{2}}\right)$ only on the transmission of HIV/AIDS in humans.

**Fig 3:**
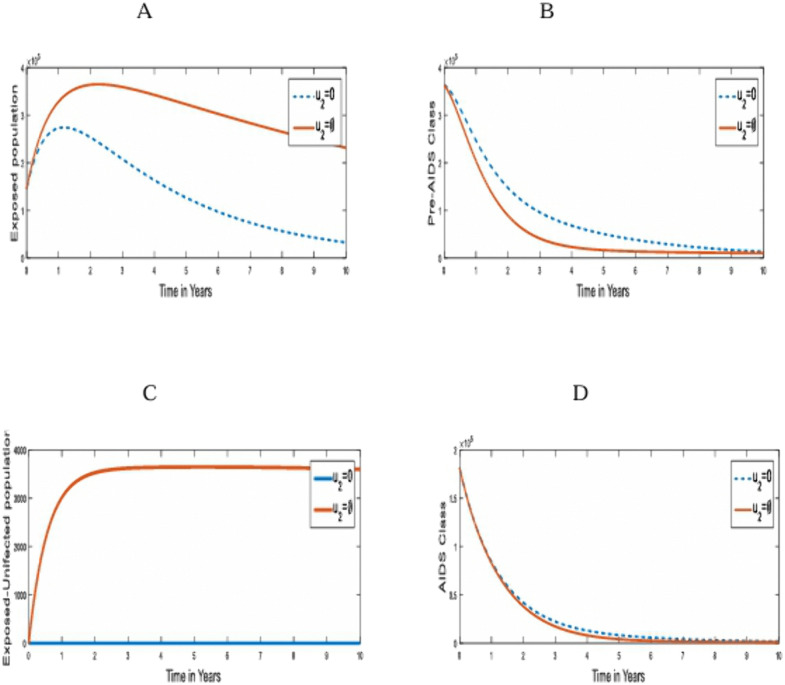
Simulations of the effect of Strategy 2 on the optimal control model. **A.** under-3-day Exposed population; **B.** Pre-AIDS population; **C.** Exposed-Uninfected population; **D.** AIDS Population for $u_{\text{1}},u_{\text{3}}=0$.

This involves the use of Post-Exposure Prophylaxis (PEP) intervention, which must be started within 72hours of exposure to HIV infection. It is aimed at preventing the under-3-day HIV exposed individuals from being infected. Fig 3 compares the population dynamics of the under-3-day exposed, Pre-AIDS, Exposed uninfected and AIDS classes on the optimal application of strategy 2 and when it is absent.

Fig 3A shows a huge increase in the population of the under-3-day Exposed Class and a decrease in the number of Pre-AIDS individuals as depicted in Fig 3B. Also, on the application of this control strategy, many HIV Exposed individuals who could have been infected progress to the Exposed-uninfected Class as seen in Fig 3C. Fortunately, this is the only strategy that guarantees progression to the Exposed-uninfected class after an effective exposure to the disease. This intervention also averted a total of 1.3518 x10^6^ infected individuals.

**Strategy 3:** The optimal control of HIV/AIDS transmission using Anti-Retroviral treatment (ART) $\left(u_{\text{3}}\right)$only on the infected humans.

**Fig 4:**
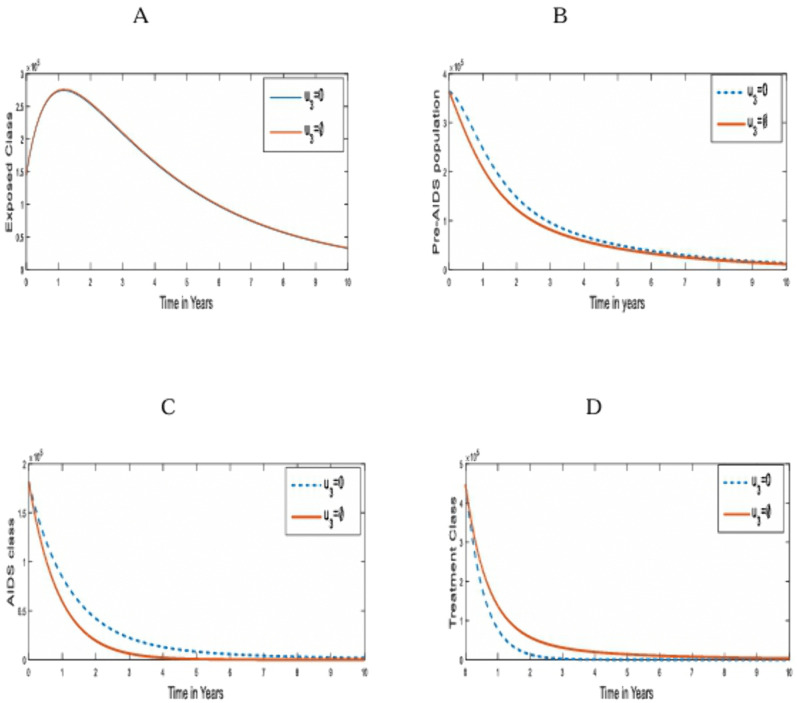
Simulations of the effect of Strategy 3 on the optimal control model. **A.** under-3-day Exposed population; **B.** pre-AIDS population; **C.** AIDS population; **D.** Treatment Population for $u_{\text{1}},u_{\text{2}}=0$.

This control measure is aimed at using anti-retroviral drugs to reduce the viral load in pre-AIDS and AIDS individuals. It is aimed at inhibiting or curtailing the transmission of the virus. Fig 4 compares the population dynamics of the under-3-day exposed, Pre-AIDS, and AIDS classes on the optimal application of strategy 3 and when it is absent.

This control strategy does not have any effect on the exposed population, as noted in Fig 4A. The control measure has a minimal effect on the Pre-AIDS population, as shown in Fig 4B. However, the control strategy averts a total of 0.4088 x10^6^ infected individuals. The strategy is most useful in averting deaths due to the infection in the AIDS population. Anti- Retroviral treatments help to reduce the viral load in the AIDS population and progress to lower viral loads of the infected population. This is shown in Fig 4C. The population of those receiving treatment rose at the implementation of this control measure.

**Strategy 4:** The optimal use of combined control Strategies: pre-contact control $\left(u_{\text{1}}\right)$ and post-contact control $\left(u_{\text{2}}\right)$ strategies on the transmission of HIV/AIDS in humans.

**Fig 5:**
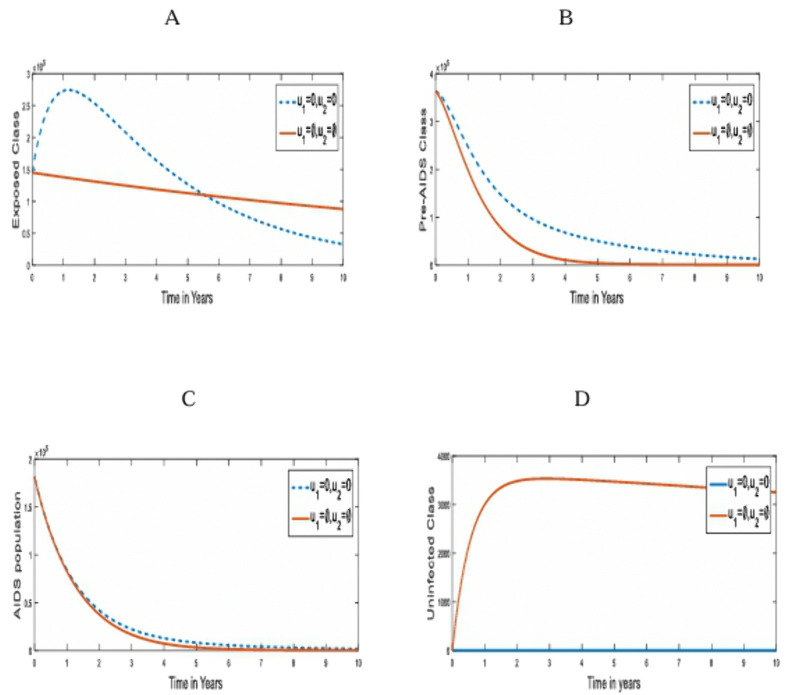
Simulations of the effect of Strategy 4 on the optimal control model. **A.** under-3-day Exposed population; **B.** pre-AIDS population; **C.** AIDS population; **D.** Uninfected Population $u_{\text{3}}=0$.

Here, we investigate the impact of the combined control strategies of pre-contact and post-contact (use of PEP) on the optimal control of HIV/AIDS infection. Fig 5 compares the population dynamics of the under-3-day exposed, Pre-AIDS; Exposed uninfected and AIDS classes on the optimal application of strategy 4 and when it is absent.

Fig 5A shows a gradual reduction in the population of the exposed. This is due to the restrictions in the population of the under 3-day-exposed because of the use of PEP, as in Fig 3A. This combined strategy promises the eradication of HIV/AIDS infection in about the 6^th^ year of its application, as seen in Fig 5B and 5C. Fig 5D shows a high uninfected rate because of PEP intervention.

**Fig 6:**
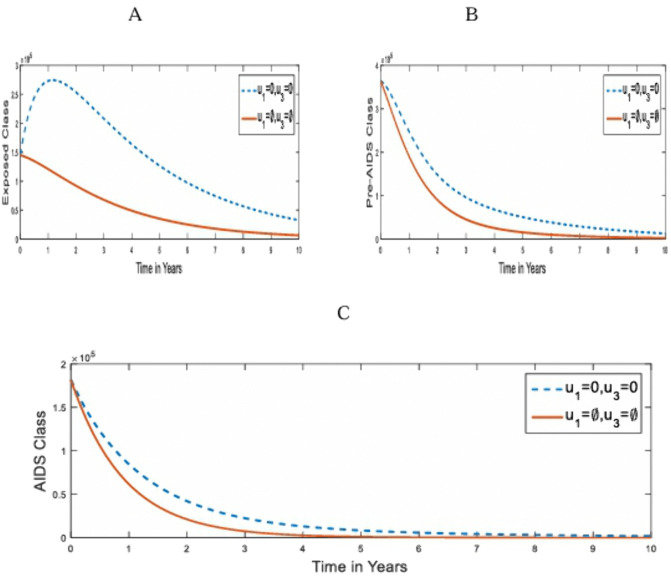
Simulations of the effect of Strategy 5 on the optimal control model. **A.** under-3-day Exposed population; **B.** Pre-AIDS population; **C.** AIDS population for $u_{\text{2}}=0$.

**Strategy 5:** The optimal use of combined control Strategies: pre-contact control $\left(u_{\text{1}}\right)$ and Treatment with ART $\left(u_{\text{3}}\right)$ strategies on the transmission of HIV/AIDS in humans. Fig 6 compares the population dynamics of the under-3-day exposed, Pre-AIDS, and AIDS classes on the optimal application of strategy 5 and when it is absent.

With this combined strategy, we notice a decline in the population of the under-3-day exposed the Pre-AIDS, and the AIDS classes as shown in Fig 6B and 6C. This is because both the HIV Pre-Contact and Treatment control measures put restrictions on them. However, no one will recover from the infection after an effective contact with the infected individual because of the absence of control strategy 2.

**Fig 7:**
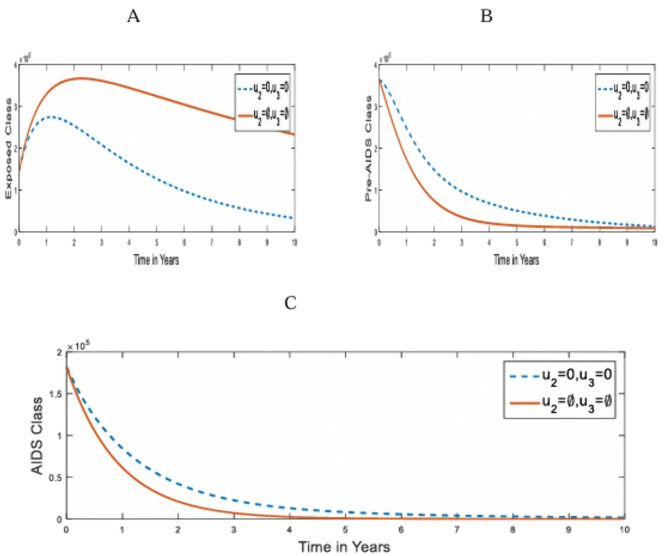
Simulations of the effect of Strategy 6 on the optimal control model. **A.** under-3-day Exposed population; **B.** pre-AIDS population; **C.** AIDS population for $u_{\text{1}}=0$.

**Strategy 6:** The optimal use of combined control Strategies: post-contact control $\left(u_{\text{2}}\right)$ and Treatment with ART control $\left(u_{\text{3}}\right)$ strategies on the transmission of HIV/AIDS in humans. Fig 7 compares the population dynamics of the under-3-day exposed, Pre-AIDS, and AIDS classes on the optimal application of strategy 6 and when it is absent.

The rise in the population of the Exposed Class is due to a lack of control measures for the susceptible class. Control with PEP intervention prevents the exposed population from contracting the infection, as shown in Fig 7A. The combined strategy also assures a low population of both the pre-AIDS and the AIDS classes but the infection will persist in the population, as shown in Fig 7B.

**Fig 8:**
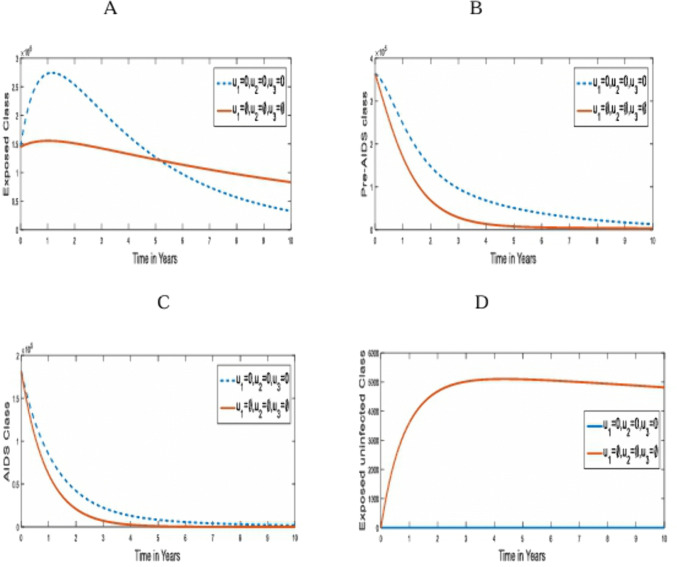
Simulations of the effect of Strategy 7 on the optimal control model. **A.** Exposed population; **B.** Pre-AIDS population; **C.** AIDS population; **D.** Exposed-Uninfected population.

**Strategy 7:** The optimal use of the three control Strategies: Pre-contact control $\left(u_{\text{1}}\right)$, post-contact control $\left(u_{\text{2}}\right)$ and Treatment with ART control $\left(u_{\text{3}}\right)$ strategies on the transmission of HIV/AIDS in humans. Fig 8 compares the population dynamics of the under-3-day exposed, Pre-AIDS, Exposed uninfected and AIDS classes on the optimal application of strategy 7 and when it is absent.

Fig 8 shows the outcome of the three combined control strategies on transmission dynamics of HIV/AIDS infection. This outcome is similar to that obtained in strategy 4, such that each of them promises the eradication of the disease from the sixth year of implementation of the control measure. Both also promise a high rate of uninfected population even after an effective exposure to the disease.

## 6. Cost-effective analysis

Here, we search for the most cost-effective and accessible control strategies that can be employed to reduce or eradicate HIV/AIDS in a given population. We investigate and compare the cost of various control strategies to find the most cost-effective of the strategies. To derive this, we use the incremental cost-effectiveness ratio (CER), which compares the differences between the costs and health outcomes of two alternative intervention strategies that compete for the same resources [15]. It is the measure of the additional cost per additional health outcome.


$$ICER=\frac{Difference\text{\textbackslash{}hspace\{0.33em}}{b}etween\text{\textbackslash{}hspace\{0.33em}\}costs\}Difference\text{\textbackslash{}hspace\{0.33em}between\text{\textbackslash{}hspace\{0.33em}\}health\text{\textbackslash{}hspace\{0.33em}\}outcomes\}$$

The number of infections averted is the difference between the number of individuals infected without the control strategy and the number infected with the control strategy. For instance, the number of individuals infected without any control strategy is 9.4088 x 10^6^, while those infected with strategy 1 control intervention is 6.9496 x 10^6^. Therefore the intervention averted 2.4592 x 10^6^ infections. Table 4 outlines the number of infections averted by each control measure, with the estimated costs.

**Table 4:** Total infection averted and the respective cost of implementing the strategies.

Control measures	Strategies	Total infection averted	Total cost	ICER
No Strategy	0	0	0	–
Single control	Strategy 1 Strategy 2 Strategy 3	2.4592 x 10^6^ 1.3518 x 10^6^ 0.4066 x 10^6^	14980 12134 13576	0 0 0
Double control	Strategy 4 Strategy 5 Strategy 6	1.7255 x 10^6^ 3.8802 x 10^6^ 2.3696 x 10^6^	46790 32680 43480	0 0 0
Triple control	Strategy 7	2.6958 x 10^6^	82755	0

For the single control strategies:

We compare strategy 1 with the next less effective alternative (strategy 2) and compute as follows:


$$\begin{array}{c} ICER\left(2\right)=\frac{12134}{1351800}=0.00898\\ ICER\left(1\right)=\frac{14980-12134}{2459200-1351800}=0.00257 \end{array}$$

This is represented in Table 5.

**Table 5:** Comparison between strategies 2 and 1.

Strategies	Total infection averted	Total cost	ICER
1 2	2.4592 x 10^6^ 1.3518 x 10^6^	14980 12134	0.00257 0.00898

Since, ICER(1)<ICER(2), We discard strategy 2 and construct Table 6 to compare strategies 1 and 3.

**Table 6:** ICER comparison of strategies 1 and 3.

Strategies	Total infection averted	Total cost	ICER
1 3	2.4592 x 10^6^ 0.4088 x 10^6^	14980 13576	0.0006847 0.0332


$$\begin{array}{c} ICER\left(3\right)=\frac{13576}{408800}=0.0332\\ ICER\left(1\right)=\frac{14980-13576}{2459200-408800}=0.0006847 \end{array}$$

ICER(1)<ICER(3), implying that strategy 3 is more expensive than strategy 1.

Hence, strategy 1 is the least expensive of the three single strategies.

For double strategies:

Comparing strategies 4 and 5, we have


$$\begin{array}{c} ICER\left(4\right)=\frac{46790}{1725500}=0.02712\\ ICER\left(5\right)=\frac{32680-46790}{3880300-1725500}=-0.006548 \end{array}$$

We represent this in Table 7.

**Table 7:** ICER comparison of strategies 4 and 5.

Strategies	Total infection averted	Total cost	ICER
4 5	1.7255 x 10^6^ 3.8803 x 10^6^	46790 32680	0.02712 −0.006548

ICER(5)<ICER(4), we discard strategy 4 and compare strategies 5 and 6.


$$\begin{array}{c} ICER\left(6\right)=\frac{43480}{2369600}=0.01835\\ ICER\left(5\right)=\frac{32680-43480}{3880300-2369600}=-0.00715 \end{array}$$

This is represented in Table 8.

**Table 8:** ICER comparison of strategies 5 and 6.

Strategies	Total infection averted	Total cost	ICER
5 6	3.8803 x 10^6^ 2.3696 x 10^6^	32680 43480	−0.00715 0.01835

ICER(5)<ICER(6), we discard strategy 6 and conclude that strategy 5 is the least expensive among the double strategies.

### 6.1 The most cost-effective strategy of all the control strategies.

We have identified that among the single strategies, strategy 1 is the most cost-effective. In double strategies, control strategy 5 is the cheapest in implementation.

To determine the most cost-effective control measures, we compare and select among strategies 1, 5, and 7.

Comparing strategy 1and 5, we have:


$$\begin{array}{c} ICER\left(1\right)=\frac{14980}{2459200}=0.00609\\ ICER\left(5\right)=\frac{32680-14980}{3880300-2459200}=0.0125 \end{array}$$

Represented in Table 9 as;

**Table 9:** ICER comparison of strategies 1 and 5.

Strategies	Total infection averted	Total cost	ICER
1 5	2.4592 x 10^6^ 3.8803 x 10^6^	14980 32680	0.00609 0.0125

ICER(1)<ICER(5), we discard strategy 5 and compare strategies 1 and 7.

Comparing strategies 1 and 7, we have:


$$\begin{array}{c} ICER\left(1\right)=\frac{14980}{2459200}=0.00609\\ ICER\left(7\right)=\frac{82755-14680}{2695800-2459200}=0.2877 \end{array}$$

Also, represented in Table 10 as;

**Table 10:** ICER comparison of strategies 1 and 7.

Strategies	Total infection averted	Total cost	ICER
1 7	2.4592 x 10^6^ 2.6958 x 10^6^	14980 82755	0.00609 0.2877

From Table 10, we notice that ICER(1)<ICER(7). Thus, we discard strategy 7 and conclude that strategy 1 is the least expensive among all the strategies in implementation.

Hence, from the cost-effectiveness analysis, the Pre-contact control measure, which involves awareness campaigns, use of condoms, PrEP, and other pre-contact preventive interventions, is the best cost-effective strategy among all the discussed control strategies.

## 7. Conclusion and recommendations

Since there is no known cure for HIV/AIDS infection yet, it becomes pertinent that multiple control interventions should be employed to the disease. To further understand the spread and control of the disease, we developed a modified mathematical model of HIV/AIDS transmission dynamics with three main strategic controls. We first presented and analyzed the basic model without optimal control variables. The reproduction number $\left(R_{e}\right)$ was obtained and used to determine the model’s stability. It was found that the disease-free equilibrium would be asymptotically stable when $R_{\varepsilon }<1$ and that the unique endemic equilibrium exists whenever $R_{\varepsilon }>1$. We then extended the basic model by incorporating three time-dependent control variables to investigate the impact of various optimum control variables. The control strategies include: Pre-contact control strategies $\left(u_{\text{1}}\right)$, which involve all preventive measures aimed at avoiding the disease contact with the susceptible population; post-contact control $\left(u_{\text{2}}\right)$, which is the use of PEP on the exposed individuals to prevent the infection; and Treatment controls $\left(u_{\text{3}}\right)$, used on the infected population to reduce the viral burden in them.

Pontryagin’s maximal principle technique was used to obtain the optimal solutions. We used the fourth-order Runge-Kutta forward-backward sweep method to simulate the dynamics of the susceptible, Exposed, Pre-AIDS, Exposed but uninfected, and AIDS populations with and without these control measures. It was found that each control strategy reduced the HIV burden of the infected population, compared to that without control, as can be seen from Fig 2 through Fig 8. It can also be seen that strategy 7, which is the combination of all the single strategies, had the highest number of infected persons averted (2.6958 x 10^6^). It is worth noting that only strategy 2, the use of PEP to control HIV infection, or its combinations with other strategies, can produce an exposed but uninfected population after an effective contact with the disease. However, the exposure must be under 3 days for it to work. Strategies 4 and 7 promise the eradication of the disease in the population within the sixth year, with a high uninfected population after exposure to the HIV infection, as shown in Figs 5 and 8. It was also deduced that strategy 1, the use of preventive measures to prevent susceptible populations’ contact with the disease, is the most economical and effective among all the strategies.

It is therefore advised that, for minimal cost, respective Governments and health policy makers, especially in sub-Saharan Africa, should invest in this strategy 1. This could be achieved through public education and awareness campaigns on the prevention of HIV infection. Though, Post-contact control strategy (use of PEP) might be costly to implement, under-3-day HIV exposed individuals are difficult to track, and the strategy is hard to execute within the required time limit; it should be aggressively pursued to prevent those accidentally exposed to the virus from contracting the infection. Hence, both PrEP and PEP drugs should be made available and affordable to the populace.
